# Cell-in-cell structure mediates in-cell killing suppressed by CD44

**DOI:** 10.1038/s41421-022-00387-1

**Published:** 2022-04-19

**Authors:** Yan Su, Hongyan Huang, Tianzhi Luo, You Zheng, Jie Fan, He Ren, Meng Tang, Zubiao Niu, Chenxi Wang, Yuqi Wang, Zhengrong Zhang, Jianqing Liang, Banzhan Ruan, Lihua Gao, Zhaolie Chen, Gerry Melino, Xiaoning Wang, Qiang Sun

**Affiliations:** 1 Beijing Institute of Biotechnology, Research Unit of Cell Death Mechanism, 2021RU008, Chinese Academy of Medical Science, 20 Dongda Street, Beijing, China; 2 CAS Key Laboratory of Mechanical Behavior and Design of Materials, Department of Modern Mechanics, University of Science and Technology of China, Hefei, China; 3 Department of Oncology, Beijing Shijitan Hospital of Capital Medical University, Beijing, China; 4 Departments of Experimental Medicine, University of Rome Tor Vergata, Rome, Italy; 5 DZNE German Center for Neurodegenerative Diseases, Bonn, Germany; 6 National Research Center of Geriatrics Diseases, Chinese PLA General Hospital, Beijing, China; 7 School of Laboratory Medicine and Biotechnology, Southern Medical University, Guangzhou, China

**Keywords:** Apoptosis, Cancer immunotherapy, Tumour immunology

## Abstract

Penetration of immune cells into tumor cells was believed to be immune-suppressive via cell-in-cell (CIC) mediated death of the internalized immune cells. We unexpectedly found that CIC formation largely led to the death of the host tumor cells, but not the internalized immune cells, manifesting typical features of death executed by NK cells; we named this “in-cell killing” which displays the efficacy superior to the canonical way of “kiss-killing” from outside. By profiling isogenic cells, CD44 on tumor cells was identified as a negative regulator of “in-cell killing” via inhibiting CIC formation. CD44 functions to antagonize NK cell internalization by reducing N-cadherin-mediated intercellular adhesion and by enhancing Rho GTPase-regulated cellular stiffness as well. Remarkably, antibody-mediated blockade of CD44 signaling potentiated the suppressive effects of NK cells on tumor growth associated with increased heterotypic CIC formation. Together, we identified CIC-mediated “in-cell killing” as a promising strategy for cancer immunotherapy.

## Introduction

Cell-in-cell (CIC) structures, characterized by the presence of one or more cells inside another cell, were prevalent in a wide range of human tumors^1,2^. The formation of CIC structures could result in the death of internalized cells, which, therefore, was proposed a novel type of programmed cell death^3^. This concept was independently confirmed in mechanistically different CIC models, such as entosis^4^, emperitosis^5^, cannibalism^6^. CIC profiling by multiplex analysis identified two major categories, including the homotypic CIC structures formed between tumor cells, and heterotypic CIC structures primarily formed by tumor cells internalizing immune cells^7^. Both homotypic and heterotypic CIC structures were shown to be associated with tumor malignancy and patient survival^8–11^. Consistently, recent studies demonstrated a pivotal role of homotypic CIC structures in mediating tumor evolution^12,13^. This process worked as a competition mechanism between tumor cells, which was genetically regulated by core machineries including adherens junction, actomyosin, and mechanical ring^14,15^ and oncogenic mutations like KRasV12, mutant p53, and CDKN2A inactivation^16–19^. Meanwhile, immune cells were found able to penetrate into tumor cells to form heterotypic CIC structures, frequently leading to the death of the internalized immune cells^20^. For example, metastatic melanoma cells could cannibalize live T lymphocytes as a way to feed^6,21^, Natural killer (NK) cells inside tumor cells may die in an apoptotic way, likely due to reuptake of the released granzyme B (GZMB), for tumor cell survival^5,22^. Therefore, it was proposed that tumor cells might take advantage of CIC-mediated inner cell death as a way of immune evasion^23,24^, which, however, is largely speculative and remains as an open question for the field yet.

To address the above question, we employed the CIC model of emperitosis, where NK cells were the effector cells internalized by tumor cells^5^, to investigate the relationship between CIC formation and immune killing, and the underlying mechanisms. Unexpectedly, we uncovered an unusual way of NK cell killing from inside of the tumor cells, which demonstrated much higher efficiency than the traditional killing from outside. Furthermore, we identified CD44, a transmembrane glycoprotein critical for tumor development and progression^25^, as a negative regulator of heterotypic CIC formation, which might be targeted for tumor immunotherapy.

## Results

### CIC structures mediate in-cell killing of tumor cells

**Fig. 1:**
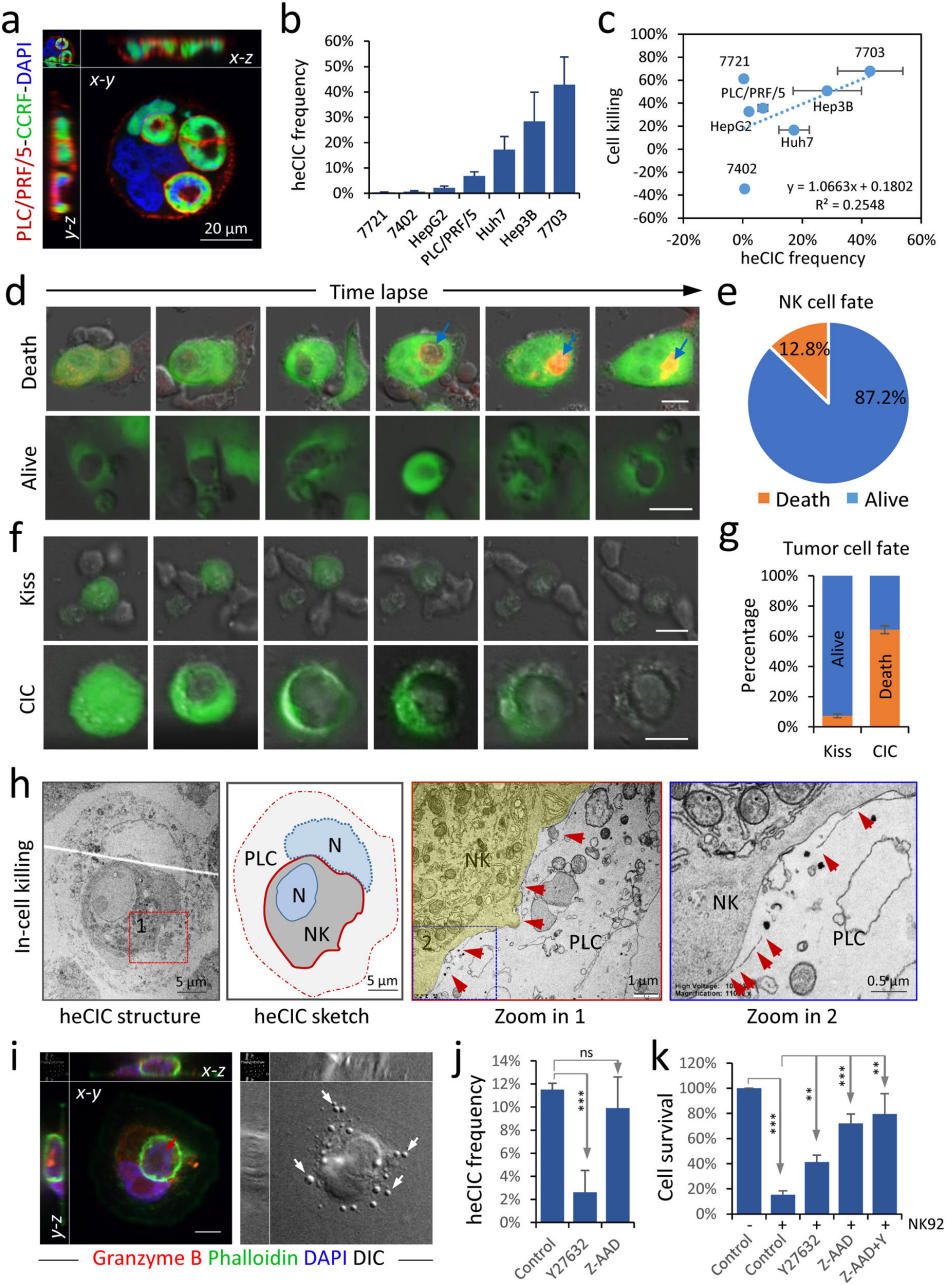
In-cell killing mediated by heterotypic cell-in-cell (heCIC) structure. **a** Representative images of heCIC structures formed between PLC/PRF/5 cell (phalloidin-red) and CCRF-CEM cells (EGFP-green). Scale bar, 20 µm. **b** The frequencies of heCIC formation in a panel of liver tumor cell lines co-cultured with CCRF-CEM cells for 8 h. *n* > 800 cells for each cell line. Data are means ± SD of quintuple experiments. **c** Correlation between heCIC frequency and immune killing efficiency in a panel of liver tumor cell lines. Data are means ± SD of triplicate experiments. **d**, **e** Fate analysis of NK92MI cells internalized into PLC/PRF/5 cells stained with lysotracker-red by time-lapse imaging of 24 h. Scale bar, 10 µm. *n* = 125. **f**, **g** Fate analysis of PLC/PRF/5 cells that were in contact with NK92MI cells from outside (kiss), or internalized NK92MI cells (CIC), by time-lapse imaging of 24 h. Scale bar, 10 µm. *n* = 80 for kiss-killing, 117 for CIC, respectively. Data are means ± SD of triplicate experiments. **h** Transmission electron microscope image for a heCIC structure with an NK92MI cell inside a dying PLC/PRF/5 cell. The zoomed images showed the broken cellular membrane (red arrows) of the dying host PLC/PRF/5 cells. Scale bars: 5 µm, 5 µm, 1 µm, and 1 µm, respectively. **i** Representative images for a heCIC structure with an NK92MI cell (red arrow) internalized by a PLC/PRF/5 cell, stained with anti-granzyme B antibody (red), phalloidin (green), and DAPI (blue). White arrows indicate blebs on PLC/PRF/5 cell. Scale bar, 10 µm. **j** The formation of heCIC structures between PLC/PRF/5 and NK92MI cells for 6 h (E: T = 4: 1), treated with DMSO, Y27632 (10 µM), and Z-AAD-CMK (50 µM), respectively, as quantified on cytospin images. Data are means ± SD of quintuple experiments. ***P < 0.001. **k** The percentage of PLC/PRF/5 cells that survived 24 h co-culture with NK92MI cells in the presence of Y27632 (10 µM), Z-AAD-CMK (50 µM), and Y27632 plus Z-AAD-CMK, respectively, quantified by way of CCK8 incorporation. Data are means ± SD of triplicate experiments. **P < 0.01; ***P < 0.001.

Since CIC formation was reported to mediate the death of internalized NK cells in emperitosis^5^, we, therefore, hypothesized that the frequency of CIC formation would negatively correlate with tumor cell killing by NK92MI cells, an immortalized NK cell line. To test this idea, a group of liver cancer cell lines were first co-cultured with CCRF-CEM, a human lymphoblast cell line defective in cytotoxic function, to access their CIC formation abilities. As shown in Fig. 1a, b and Supplementary Fig. S1a, the liver cancer cells could internalize CCRF-CEM cells at different frequencies, with the lowest in SMMC-7721 and BEL-7402 cells and the highest in QGY-7703 cells. We then examined their killing by NK92MI cells. Unexpectedly, the killing efficiencies were positively correlated with cells’ abilities to form CIC structures (Fig. 1c), suggesting that CIC formation may, instead of inhibiting, facilitate tumor cell killing by NK cells.

To address this issue, time-lapse imaging was performed on CIC structures formed between NK92MI and PLC/PRF/5, a hepatocellular carcinoma cell with intermediate CIC frequency to allow capture of sufficient CIC structures. As shown in Fig. 1d, we did observe the death of internalized NK92MI cells along with positive staining of lysotracker-red, however in low frequency of <13% (Fig. 1e) during the period of 24 h; rather, we found that most of the outer PLC/PRF/5 cells (>60%) died as evidenced by typical morphological changes and losing the expression of EGFP, and the death rate was much higher than that (~8%) from contact-mediated kiss-killing from outside of tumor cells (Fig. 1f, g). The dominant efficiency over that of kiss-killing by CIC-mediated killing was maintained at a lower effector/target (E/T) ratio, a condition when the kiss-killing was minimally effective (Supplementary Fig. S1b). To differentiate from the traditional kiss-killing, we named this unique CIC-mediated killing as “in-cell killing”.

### Granzyme B is involved in CIC-mediated in-cell killing

Consistent with morphological changes of in-cell killing in time-lapse imaging, transmission electron microscopy (TEM) showed that the outer tumor cell that enclosures a NK92MI cell displayed typical features of cell death, characterized by disconnected plasma membrane, degenerated nucleus and organelles, and loss of cytosolic components (Fig. 1h). Since granzyme B (GZMB) is the major effective molecule of NK cells that induces target cell death, we therefore explore its involvement in the in-cell killing. Immunostaining showed that, in contrast to GZMB-negative cells, the GZMB-positive cells frequently displayed membrane blebbing, a morphological feature of activated apoptosis^26^ (Supplementary Fig. S1c, d). Importantly, the outer cells with membrane blebs were also positive in GZMB (Fig. 1i and Supplementary Fig. S1c), suggesting the GZMB might be responsible for the death of outer tumor cells. To test this idea, we treated the co-culture with Z-AAD-CMK, a selective GZMB inhibitor, which significantly increased PLC/PRF/5 cell survival (Fig. 1k) and reduced the death of PLC/PRF/5 cells by either “in-cell killing” or “kiss-killing” (Supplementary Fig. S1l). This effect was not a secondary effect from CIC inhibition as did by Y27632, an inhibitor of RhoA signaling that is essential for active cell penetration, as Z-AAD-CMK treatment had little effect on CIC formation (Fig. 1j and Supplementary Fig. S1e, k), and the inhibited PLC/PRF/5 death by Y27632 was correlated with reduced CIC formation and CIC-mediated cell death (Supplementary Fig. S1i–k). Whereas, inhibition of either CIC formation or GZMB activation significantly attenuated target cell apoptosis as evidenced by the inhibited processing of caspase 3, the key mediator of apoptotic cell death (Supplementary Fig. S1f–h). Together, the above data support that GZMB is involved in the CIC-mediated in-cell killing.

### CD44 on tumor cells negatively regulates heterotypic CIC formation

**Fig. 2:**
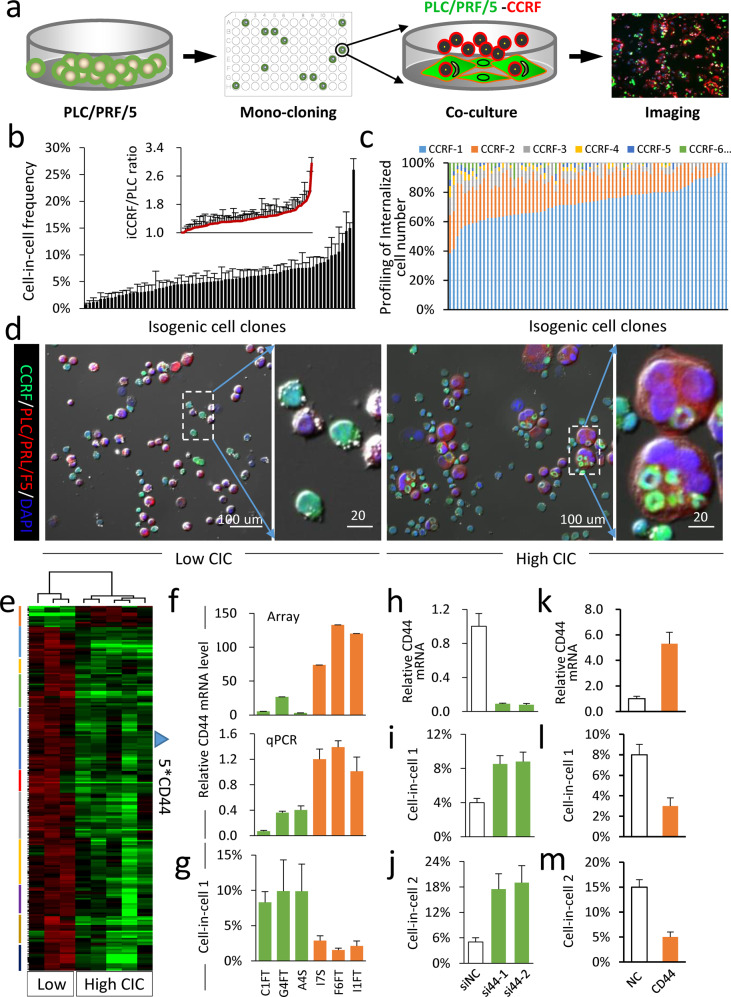
Identification of CD44 as a negative regulator of heCIC formation. **a** Schematic diagram of isolating isogenic clones from PLC/PRF/5 by limited dilution for heCIC assay. **b**, **c** HeCIC profiling for the isogenic clones, displayed as heCIC frequency (**b**), average number of internalized CCRF-CEM cells (the insert graph of **b**), and the percentage of heCIC structures with different internalized CCRF-CEM cells (**c**). Data are means ± SD of quantifications from more than two independent investigators. More than three images of 20× objective were quantified for each isogenic clone. **d** The representative images of cytospins for isogenic clones of low and high heCIC formation. Scale bars: 100 μm, and 20 µm, respectively. **e** The unsupervised clustering of genes differentially expressed between three low-CIC and five high-CIC isogenic clones, and green for low expression, red for high expression. Five probes for CD44 were identified in the gridded region. **f** Relative mRNA level of CD44 by mRNA microarray and quantitative real-time PCR (RT-PCR) in PLC/PRF/5 isogenic clones. **g** HeCIC formation in isogenic cell clones co-cultured with CCRF-CEM cells. Data are means ± SD of more than three images of 20× objective. *n* > 300 cells analyzed for each clone. **h** Relative expression of CD44 upon small interfering RNA (siRNA)-mediated knockdown in F6ft cells. Data are means ± SD of triplicate experiments. **i**, **j** Increased formation of heCIC structures in control and CD44-depleted PLC/PRF/5 cells. Cell-in-cell 1 (%) = numbers of PLC/PRF/5 cells in CICs/number of total PLC/PRF/5 cells; Cell-in-cell 2 (%) = numbers of internalized CCRF cells/number of total PLC/PRF/5 cells counted. Data are means ± SD of more than three images of 20× objective. *n* > 300 cells analyzed for each clone. **k**–**m** Overexpression of CD44 (**k**) inhibits heCIC formation (**l**, **m**) in A4S cells. Data are means ± SD of more than three images of 20× objective. *n* > 300 cells analyzed for each cell.

Since CIC formation is conceivably regulated by the active interactions between NK and tumor cells, we set out to explore the molecules on tumor cells that might regulate the formation of heterotypic CIC structures. We first managed to isolate 74 isogenic clones from the PLC/PRF/5 cells by limited dilution and then examined their abilities to form heterotypic CIC structures with EGFP-expressing CCRF-CEM cells by co-culture assay (Fig. 2a). Interestingly, these isogenic clones displayed a wide range of CIC formation frequency with a scope from about 1% to 25% (Fig. 2b–d), and it is common that one tumor cell internalized multiple CCRF-CEM cells (Fig. 2c), up to >9 CCRF-CEM cells could be found in one PLC/PRF/5 cell (Supplementary Fig. S2a) with 2.95 CCRF-CEM per PLC/PRF/5 as the highest average level (insert graph of Fig. 2b).

The inter-clonal difference in CIC formation frequency allowed us to identify genes regulating CIC formation by expression profiling analysis. Based on the CIC frequency, totally eight clones (three low and five high) were selected for microarray-based mRNA profiling (Fig. 2d, e and Supplementary Fig. S2b), which reported CD44 as a candidate gene that expressed at relatively low level in clones with high-CIC frequency. The differential expression of CD44 was confirmed by quantitative RT-PCR in six isogenic clones (C1FT, G4FT, and A4S for high-CIC group, I7S, F6tf, and I1FT for low-CIC group) (Fig. 2f, g). To establish a functional link to CIC formation, CD44 was knocked down in F6ft cells by siRNAs targeting two different sites, which resulted in an increased formation of CIC structures (Fig. 2h–j and Supplementary Fig. S2c). Whereas, ectopic overexpression of CD44 significantly inhibited CIC formation (Fig. 2k–m and Supplementary Fig. S2d). Together, we identified CD44 as a negative regulator of heterotypic CIC formation on tumor cells.

### CD44 inhibits N-cadherin-mediated intercellular adhesion

**Fig. 3:**
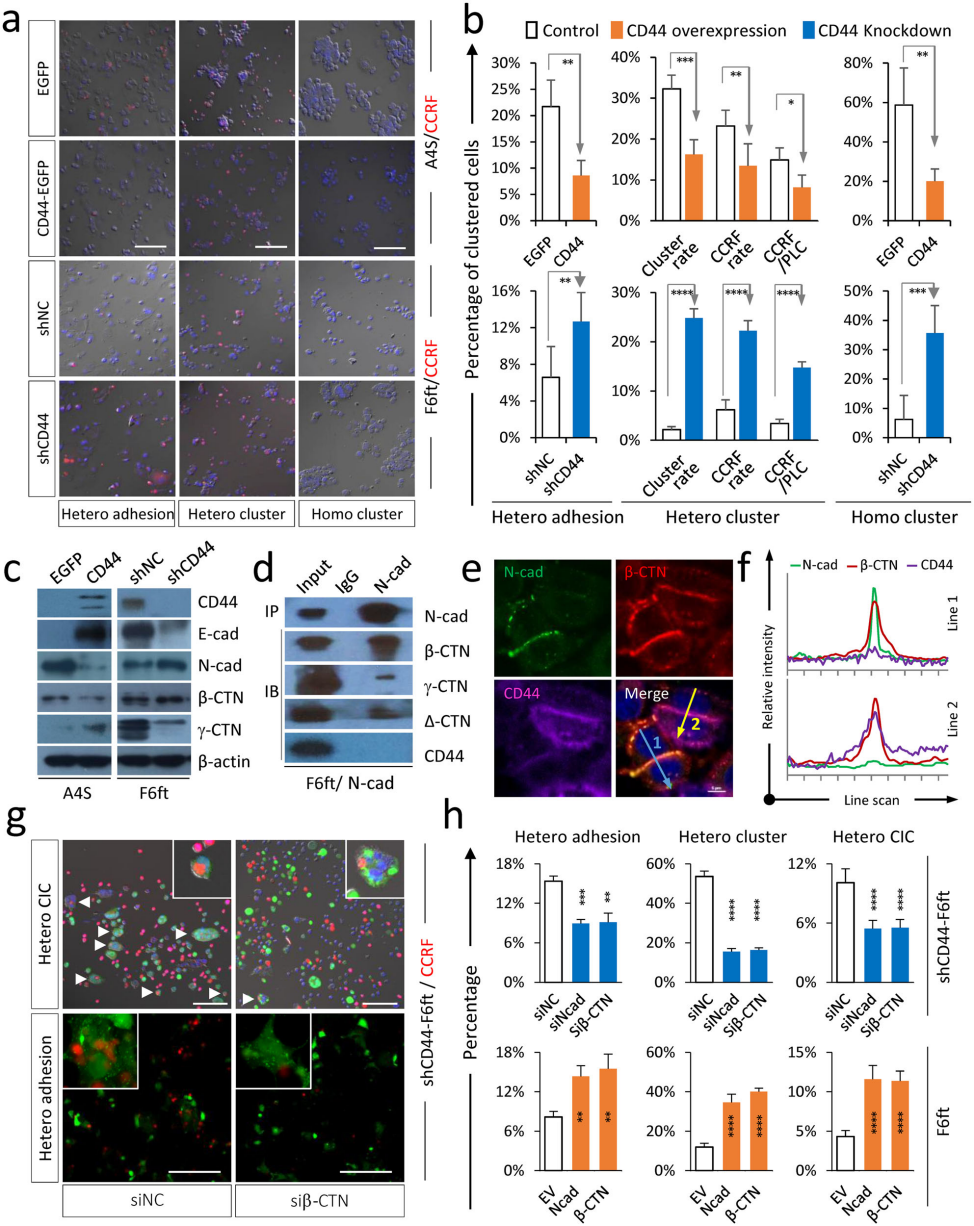
CD44 inhibited intercellular adhesion by downregulating N-cadherin and β-catenin expression. **a**, **b** Representative images (**a**), and quantification (**b**) of heterotypic intercellular adhesion (hetero adhesion), heterotypic cell clustering (hetero cluster) formed between CCRF-CEM (red) and isogenic cells, and homotypic cell clustering (homo cluster) formed between isogenic tumor cells. Heterotypic adhesion = CCRF-CEM adhered to PLC/PRF/5/total PLC/PRF/5 cells. For heterotypic cluster, the cluster rate = cells in cluster/total cells, the CCRF rate = CCRF-CEM in cluster/total CCRF-CEM, and the CCRF/PLC = CCRF-CEM in cluster/total PLC/PRF/5. Homotypic cluster = percentage of PLC/PRF/5 cells in cluster. The cell cluster was defined as a cell colony that contains >6 cells. Scale bar = 50 μm. Data are means ± SD of six images of 20× objective. *n* > 1000 cells analyzed for each cell. *P < 0.05; **P < 0.01; ***P < 0.001; ****P < 0.0001. **c** Expression of adhesion molecules upon CD44 overexpression or CD44 knockdown detected by Western blot. The β-actin was used as a loading control. **d** Interaction between N-cadherin and β-catenin analyzed by co-immunoprecipitation blot. **e** Representative images depict the subcellular localization of N-cadherin (green), β-catenin (red), and CD44 (purple) in PLC/PRF/5 cells by confocal microscopy. Scale bar, 10 µm. **f** The relative fluorescent intensity on line 1 and line 2 in **e** quantified by line scanning. **g** Images showing heterotypic intercellular adhesion (hetero adhesion) and heterotypic cell clustering (hetero cluster) formed between CCRF-CEM (red) CD44-depleted F6ft cells with β-catenin co-depletion. Scale bars, 50 μm. **h** N-cadherin and β-catenin mediate enhanced heterotypic intercellular adhesion (hetero adhesion), heterotypic cell clustering (hetero cluster), and heterotypic CIC formation (hetero CIC) upon CD44 depletion. Data are means ± SD of six images of 20× objective. *n* > 1000 cells analyzed for each cell. **P < 0.01; ***P < 0.001; ****P < 0.0001.

Cell–cell adhesion is known to be critical for homotypic CIC formation^18^, we therefore investigated the influence of CD44 on intercellular adhesion. As shown in Fig. 3a, b and Supplementary Fig. S2e, f, ectopic expression of CD44 in A4S cells (low CD44; high CIC) inhibited their adhesion with CCRF-CEM cells in co-culture assay (hetero adhesion), which was unlikely to be the artificial effects from matrix adhesion as the heterotypic cluster formation was also compromised in suspension culture (hetero cluster). This effect was associated with reduced adhesive ability of A4S cells upon CD44 overexpression as evidenced by reduced formation of homotypic cluster in suspension (homo cluster). The inhibitory effects of CD44 on cell adhesion were confirmed in F6ft cells with CD44 depletion, where intercellular adhesions were significantly enhanced (lower panels in Fig. 3a, b).

We next explored the molecular changes underlying CD44-inhibited cell adhesion by examining the expression of E-cadherin, an adhesive molecule that was reported to be essential for the formation of both homotypic and heterotypic CIC formation^14,27^. However, E-cadherin was unlikely to be responsible for the compromised adhesion by CD44 because it was positively regulated by CD44 (Fig. 3c and Supplementary Fig. S3a), and the reason that upregulated E-cadherin failed to enhance intercellular adhesion might be ascribed to its primary cytosolic localization (Supplementary Fig. S3b), the same was true for γ-catenin that was primarily in complexed with E-cadherin (Supplementary Fig. S3c, d). On the contrary, N-cadherin, in complex largely with β-catenin (Fig. 3d and Supplementary Fig. S3e), was negatively regulated by CD44 and primarily localized at the intercellular contacts in a pattern complementary to CD44 expression (Fig. 3c–f), and was more likely responsible for CD44’s effects on cell adhesion. Consistent with which, knocking down either N-cadherin or β-catenin significantly compromised cell–cell adhesion, cluster formation, and CIC formation in CD44-depleted cells, and their ectopic expression promoted the formation of heterotypic CIC structures that was associated with enhanced cell–cell adhesion and cluster formation in CD44-high F6ft cells (Fig. 3g, h; Supplementary Fig. S3g–i). Together, these data support the involvement of downregulated N-cadherin/β-catenin and compromised cell–cell adhesion in CD44-inhibited CIC formation.

### CD44 enhances Rho GTPase-regulated cellular stiffness

**Fig. 4:**
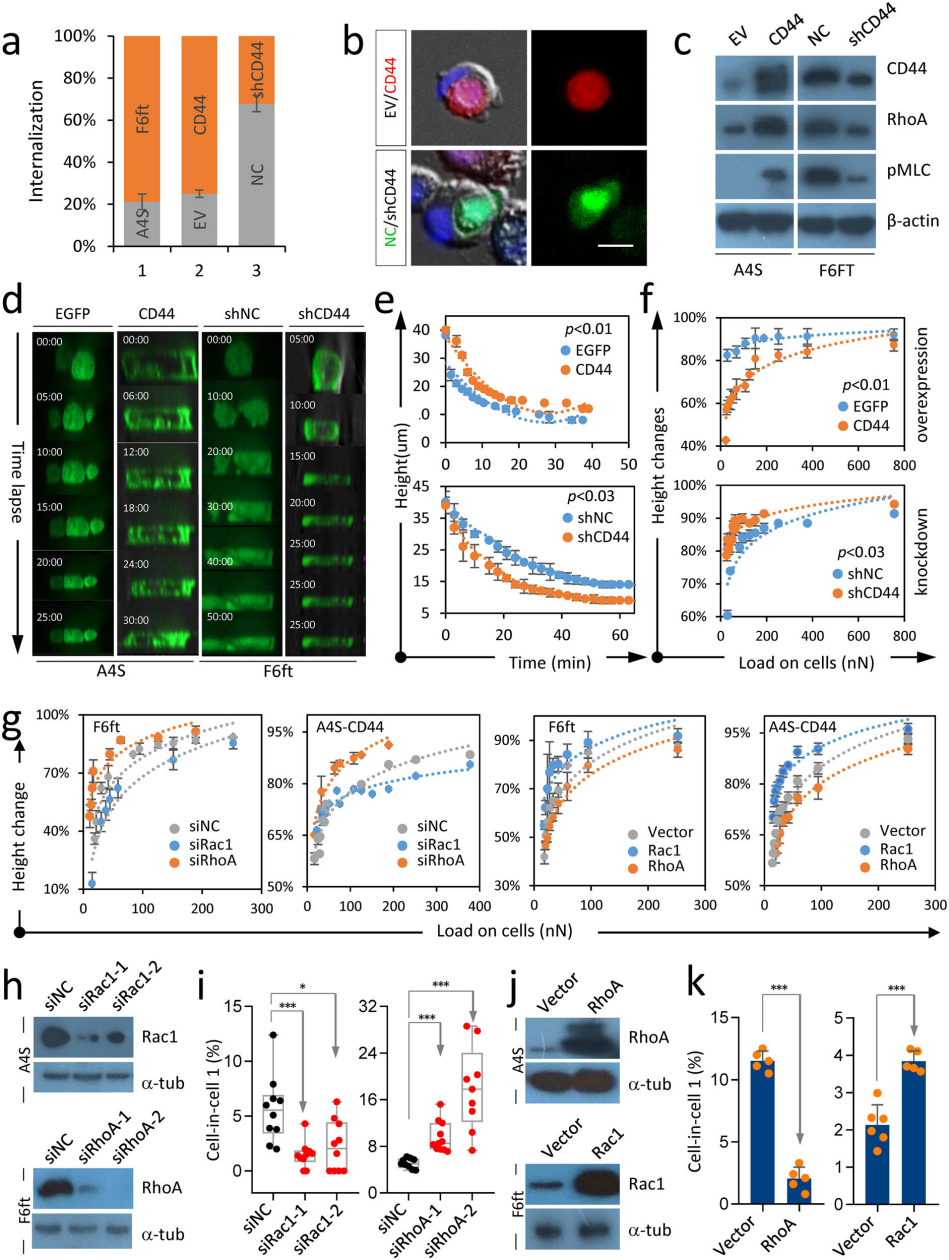
CD44 enhanced cell rigidity via RhoA and Rac1. **a** CD44 promoted cell internalization. Orange and gray represented the internalized cells with relatively high and low CD44 expression, respectively. EV: empty vector, NC: non related control. Data are means ± SD of more than three images of 20× objective. *n* > 59/each. **b** Representative images showing homotypic CIC structures with CD44-high cells internalized (in red and green, respectively). Scale bar, 20 µm. **c** CD44 positively regulated the expression of RhoA and pMLC in PLC/PRF/5 isogenic cells. **d** Time-lapse images in *x*–*z* plane for the deformation of PLC/PRF/5 isogenic cells compressed by agarose gel. **e**, **f** CD44 regulated cellular deformation dynamics over time (**e**), or in response to different weight loads (nN) (**f**) as determined by agarose compression assay. Data are means ± SD of triplicate experiments. **g** CD44 enhanced cell rigidity via RhoA and Rac1 as determined by agarose compression assay. Data are means ± SD of triplicate experiments. **h** The siRNA-mediated knockdown of Rac1 and RhoA in A4S and F6ft cells, respectively. **i** Formation of heCIC structures were inhibited, or promoted by knocking down Rac1 or RhoA, respectively, in PLC/PRF/5 isogenic cells. Data are means ± SD of more than three images of 20× objective. *n* > 300 cells analyzed for each clone. *P < 0.05; ***P < 0.001. **j** Overexpression of RhoA and Rac1in A4S and F6ft cells, respectively. **k** Formation of heCIC structures were inhibited, or promoted by overexpressing RhoA or Rac1, respectively, in PLC/PRF/5 isogenic cells. Data are means ± SD of more than three images of 20× objective. *n* > 300 cells analyzed for each clone. ***P < 0.001.

The formation of homotypic CIC structures was controlled by cellular rigidity, where low stiffness in the receiving/outer cells facilitate cell penetration^12^. We, therefore, asked whether CD44 in tumor cells would regulate cellular stiffness to inhibit heterotypic CIC formation. In a competition assay where cells were co-cultured in suspension, we found that F6ft cells (high CD44) preferentially penetrated into A4S (low CD44) cells; meanwhile, CD44 overexpression promoted, while CD44 knockdown reduced, cell internalization (Fig. 4a, b), suggesting that CD44 may enhance cell stiffness. Consistent with this, CD44 positively regulated RhoA signaling, a key dictator of cell rigidity^12^, as evidenced by enhanced expression of RhoA GTPase and phosphorylated myosin light chain (pMLC) upon CD44 overexpression (Fig. 4c). For further confirmation, we employed a compression assay to directly measure cell rigidity^28^, where a defined amount of agarose was overlaid onto cells. As compared with control cells, CD44-overexpressing cells were more resistant to agarose compression, and CD44-depleted cells were more easily to be deformed (Fig. 4d–f and Supplementary Fig. S4a), indicating that CD44 could increase cellular stiffness.

Cytoskeletal actomyosin was the key controller of cell rigidity and was regulated by RhoA and Rac1. These two small GTPases, working oppositely in competitive engulfment^12^, were the downstream targets of CD44^29^. We, therefore, hypothesized that RhoA and Rac1 may function downstream of CD44 to regulated cellular stiffness and heterotypic CIC formation. As shown in Fig. 4g and Supplementary Fig. S4b, c, RhoA knockdown increased cellular deformability in F6ft (CD44 high), and reversed CD44-enhanced stiffness in A4S cells; conversely, overexpressing RhoA strengthened cells’ rigidity in A4S (CD44 low), and rescued the compromised cellular stiffness resulted from CD44 depletion in F6ft cells. Whereas, Rac1 worked in a way opposite to RhoA. Importantly, RhoA depletion or Rac1 overexpression could significantly increase, while RhoA overexpression or Rac1 depletion significantly inhibited, heterotypic CIC formation (Fig. 4h–k and Supplementary Fig. S4b, e). It is noted that full-length CD44 seemed to be required in this context as truncation of either its extracellular or intracellular domain had little impact on cell–cell adhesion, cellular stiffness, or heterotypic CIC formation as well (Supplementary Fig. S5). Together, these data are consistent with a role of RhoA and Rac1, downstream of CD44, in regulating cellular stiffness and the formation of heterotypic CIC structures.

### CD44 suppresses CIC-mediated in-cell killing

**Fig. 5:**
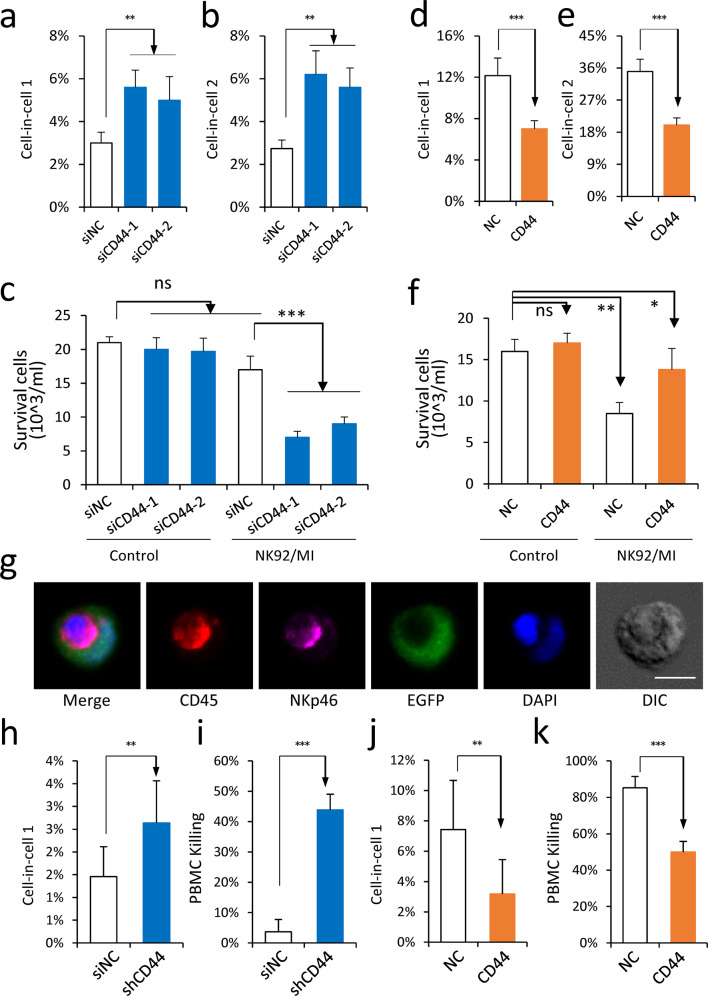
CD44-inhibited heCIC formation and immune killing. **a**, **b** CD44 knockdown in F6ft cells promoted NK92MI cell penetration to form heCIC structures (E/T = 1:1, 8 h co-culture). Data are means ± SD of more than three images of 20× objective. *n* > 300 cells analyzed for each clone. **P < 0.01. **c** CD44 knockdown in F6ft cells potentiated tumor cell killing by NK92MI cells (E/T = 1:1, 24 h co-culture) quantified by counting using a hemocytometer. Data are means ± SD of triplicate experiments. ***P < 0.001; ns, not significant. **d**, **e** Overexpression of CD44 in A4S cells NK92MI cell penetration to form heCIC formation (E/T = 1:1, 8 h co-culture). Data are means ± SD of more than three images of 20× objective. *n* > 300 cells analyzed for each clone. ***P < 0.001. **f** CD44 overexpression in A4S cells inhibited tumor cell killing by NK92MI cells (E/T = 1:1, 24 h co-culture) quantified by counting using a hemocytometer. Data are means ± SD of triplicate experiments. *P < 0.05, **P < 0.01; ns, not significant. **g** Representative images of heCIC structure formed between A4S cells (green) and PBMC-derived NK cells stained by antibodies against CD45 (red) and NKp46 (purple). Scale bar, 10 µm. **h**, **i** CD44 knockdown promoted heCIC formation (8 h co-culture) and tumor cell killing by PBMC-derived NK cells (24 h co-culture), (E/T = 1:1) quantified by counting using a hemocytometer. Data are means ± SD of triplicate experiments. **P < 0.01, ***P < 0.001. **j**, **k** CD44 overexpression inhibited heCIC formation (8 h co-culture) and tumor cell killing by PBMC-derived NK cells (24 h co-culture), (E/T = 1:1) quantified by counting using a hemocytometer. Data are means ± SD of triplicate experiments. **P < 0.01, ***P < 0.001.

Since the formation of CIC structures is essential for in-cell killing, we next explored the impacts of CD44 expression on tumor cell killing by NK cells. Indeed, we found that CD44 knockdown significantly increased formation of CIC structures between tumor and NK cells (Fig. 5a, b), and concomitantly reduced the survival of PLC/PRF/5 cells that were co-cultured with NK92MI cells (Fig. 5c and Supplementary Fig. S6a); conversely, overexpression of CD44 significantly reduced CIC formation (Fig. 5d, e) and rescued tumor cells from immune killing by NK92MI cells (Fig. 5f and Supplementary Fig. S6b). This result was also validated by utilizing NK cells derived from peripheral blood mononuclear cells (PBMC) as the killer source, where cells positive in CD45 and NKp46 could efficiently penetrate tumor cells to form CIC structures (Fig. 5g) and killed tumor cells in a CD44-dependent way that was associated with CIC formation (Fig. 5h–k). These data are consistent with a model where CD44 promotes tumor cell survival by counteracting in-cell killing.

### Antibody-mediated CD44 blockade potentiates anti-tumor activities of NK cells

**Fig. 6:**
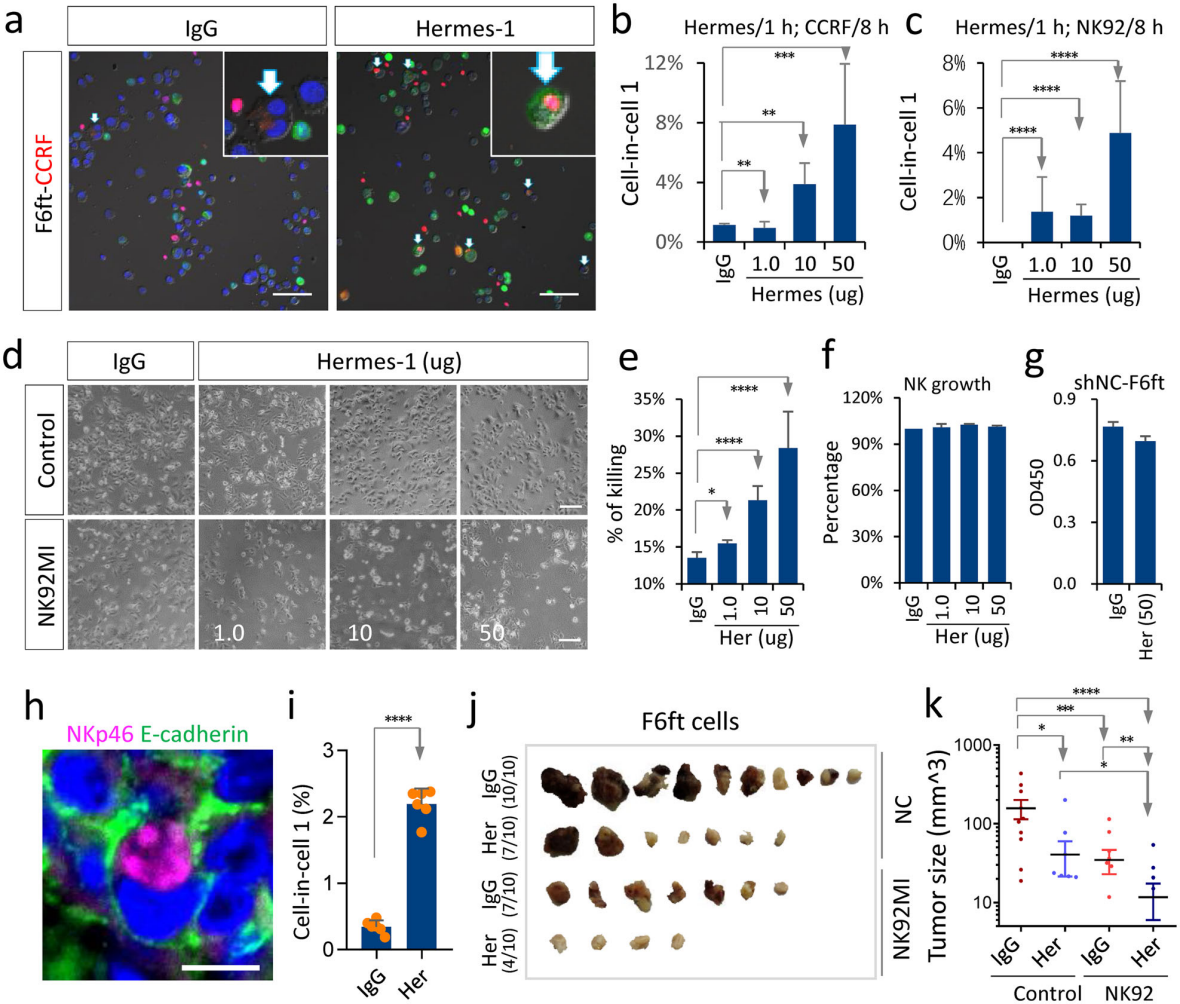
Antibody-mediated blocking of CD44 increases heCIC formation and immune killing. **a** Representative cytospin images for heCIC formation (arrows) in F6ft (green)—CCRF-CEM (red) co-culture treated with control IgG or Hermes-1, a CD44 blocking antibody. Scale bar, 50 µm. **b**, **c** Quantification of heCIC formation between F6ft and CCRF-CEM (**b**), or NK92MI (**c**). F6ft cells were pretreated with Hermes-1 for 1 h followed by co-culture for 8 h with either CCRF-CEM cells or NK92MI cells. Data are means ± SD of six images of 20× objective. *n* > 800 cells analyzed for each cell. **P < 0.01, ***P < 0.001, ****P < 0.0001. **d** Representative images for tumor cell killing by NK92MI cells in the presence of different amounts of Hermes-1 antibody. Scale bar, 50 µm. **e** Tumor cell killing by NK92MI cells was promoted by Hermes-1 (Her) treatment. Cells were co-cultured for 8 h (E/T = 4:1) before measured by Cell Counting Kit-8. *P < 0.05; ****P < 0.0001. Data are means ± SD of triplicate assays. **f**, **g** Hermes-1 (Her) treatment had little influence on the autonomous cell growth of either NK92MI (**f**) or F6ft cells (**g**). **h** Representative images of heCIC structures formed between F6ft cells and NK92MI cells in xenograft tumor tissues stained with antibodies against E-cadherin (green) and NKp46 (red). Scale bar, 15 µm. **i** Increased heCIC formation in xenograft tumor tissue treated with Hermes-1. Data are means ± SD of six images of 20× objective. ****P < 0.0001. **j** Four groups of tumors xenografts collected from the SCID Beige mice. Her: Hermes-1. **k** Quantification of xenografted tumors 21 days post the inoculation of F6ft cells together with the indicated antibodies and NK92MI (NK92) cells. Her: Hermes-1. *P < 0.05; **P < 0.01; ***P < 0.001; ****P < 0.0001. *F* test was employed.

Next, we examined the effect of Hermes-1, an anti-CD44 blocking antibody raised from rat, on tumor cell killing by NK cells. The treatment of the CD44-high F6ft cells with Hermes-1 significantly increased their abilities to internalize both CCRF-CEM and NK92MI cells to form CIC structures, which were positively correlated with the amount of antibody used (Fig. 6a–c and Supplementary Fig. S6c, d). The mechanisms underlying the enhanced CIC formation by Hermes-1 largely resembled those of CD44 depletion by RNA interference, as evidenced by enhanced cell–cell adhesion, reduced cellular rigidity, and upregulated expression of N-cadherin, β-catenin, and Rac1, though some other molecules were expressed in a different pattern (Supplementary Fig. S6f–h). In parallel with the increased CIC formation, the killing efficiency of NK cells against F6ft cells was significantly potentiated by Hermes-1 treatment in a dose-dependent manner (Fig. 6d, e and Supplementary Fig. S6e). This was unlikely to be attributed to the autonomous cell proliferation of F6ft as well as NK cells, on which Hermes-1 treatment had little effects (Fig. 6f, g), and antibody-dependent cellular cytotoxicity as Hermes-1 antibody was produced from rat^30^. To confirm the anti-tumor effects of Hermes-1 in vivo, the immune-deficient SCID/Beige mice inoculated with CD44-high F6ft cells together with Hermes-1 and NK92MI cells, which resulted in significantly increased formation of CIC structures in tumor tissues (Fig. 6h, i), and importantly a profound suppression of tumor growth, which is stronger than those imposed from either Hermes-1 or NK92MI cells alone, and IgG-treated control as well (Fig. 6j, k and Supplementary Fig. S7a, b). This effect was also validated in A4S cells ectopically expressing CD44 (Supplementary Fig. S7c–f). Together with the afore-demonstrated results, we proposed that CD44 was a potential target for tumor immune therapy through in-cell killing.

### Heterotypic CIC formation inversely associated with CD44 expression and patient survival

**Fig. 7:**
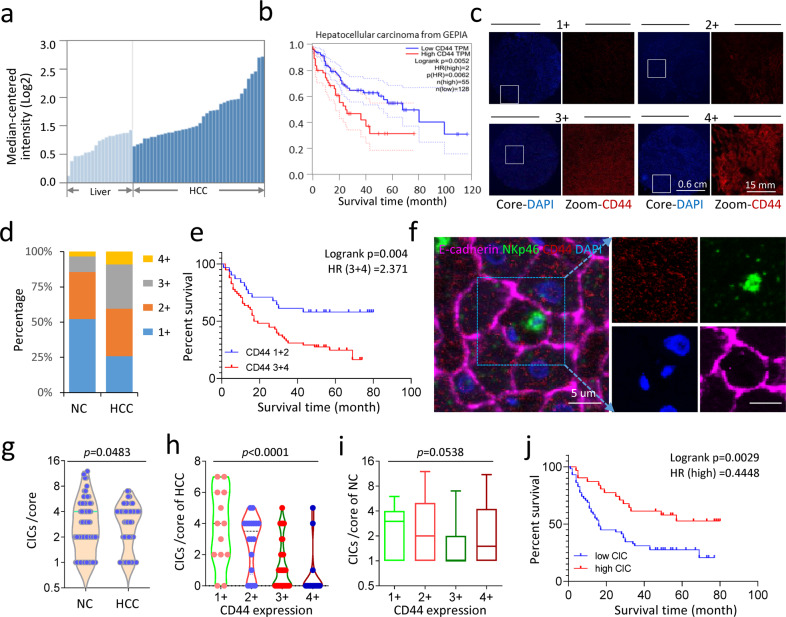
CD44 expression in tumor tissues is negatively associated with heCIC formation and patient survival. **a** The expression of CD44 mRNA in HCC tissues was higher than that in non-cancerous liver tissue. Data were retrieved from the Oncomine database. **b** High CD44 expression is associated with shorter HCC patient survival by Kaplan–Meier plotting of data from the GEPIA database. HR = 2, *P* = 0.0052, *n* = 183. **c** Representative images for four levels of CD44 expression in HCC samples of tissue microarray (TMA) by immunofluorescent staining. **d** Quantification of CD44 expression in HCC and non-cancerous (NC) liver tissues of TMA. *n* = 180 with 90 for NC and 90 for HCC, respectively. **e** High CD44 expression (3 + 4) is associated with shorter HCC patient survival by Kaplan–Meier plotting of data from TMA staining. HR = 2.371, *P* = 0.004, *n* = 90. **f** Representative images of heCIC structure formed in human HCC tissue. Scale bar, 5 µm. **g** Quantification of heCIC structures in HCC and non-cancerous (NC) liver tissues of TMA. *n* = 180 with 90 for NC and 90 for HCC, respectively. **h**, **i** CD44 expression is significantly correlated with heCIC formation in HCC (**h**), but not that in non-cancerous (NC) (**i**) liver tissues of TMA. **j** High heCIC formation (>2/core) is associated with longer HCC patient survival by Kaplan–Meier plotting of data from TMA staining. HR = 0.4448, *P* = 0.0029, *n* = 90.

To make a physiological relevance to our finding, we first analyzed the expression of CD44 in hepatocellular carcinoma (HCC) by exploring the public TCGA database, which reported a relatively higher level of CD44 mRNA expression in the cancerous tissues as compared with that in the adjacent non-cancerous (NC) tissues (Fig. 7a). Moreover, higher CD44 mRNA expression was significantly associated with unfavored survival of patients with HCC according to the analysis from GEPIA (Fig. 7b) as well as KM-plotter (Supplementary Fig. S7g)^31,32^. We then performed immunostaining on human HCC tissue microarray (TMA) to establish a link to CIC formation. Consistent with the result from public databases, the expression of CD44 protein was also higher in HCC tissues than that in the adjacent NC tissues (Fig. 7c, d), and associated with shorter patient survival (Fig. 7e and Supplementary Fig. S7h). Meanwhile, less heterotypic CIC structures were identified in HCC tissue (Fig. 7f, g). Interestingly, CD44 expression was inversely correlated with the formation of CIC structures in HCC tissues (Fig. 7h) as expected, but not those in the adjacent NC tissues (Fig. 7i). Importantly, on the contrary to CD44 expression, the high-CIC formation was positively correlated with longer patient survival (Fig. 7j). Thus, these data are in line with the above experimental results, supporting an immune-proficient role of in-cell killing suppressed by CD44 in tumor cells.

## Discussion

**Fig. 8:**
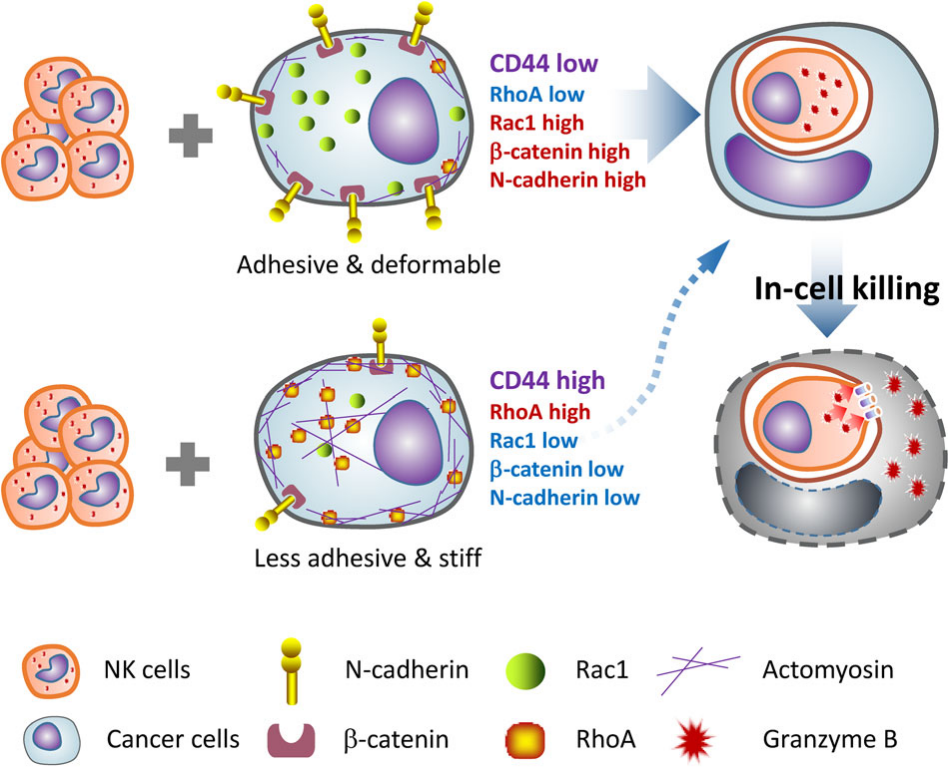
Schematic demonstration of in-cell killing regulated by CD44. When CD44 is expressed at low level (upper panel), tumor cells express low level of RhoA and high level of Rac1, N-cadherin, and β-catenin, and are therefore more adhesive and deformable to internalize NK cells for CICs-mediated in-cell killing; whereas, when CD44 is expressed at high level (lower panel), leading to RhoA upregulation and the downregulation of Rac1, N-cadherin and β-catenin as well, tumor cells become less adhesive and stiffer which inhibits NK cell internalization and the subsequent in-cell killing.

In this study, we reported in-cell killing as a novel way for immune cells to eliminate tumor cells. Distinct from the conventional kiss-killing from outside of the target cells, the more efficient in-cell killing was mediated by the penetration of NK cells into target cells to form the heterotypic CICs (Fig. 8). This finding uncovered a heretofore poorly defined function of CICs: mediating the death of outer host cells other than the inner engulfed cells. Importantly, by systemic expression profiling of isogenic cell clones that exhibited different capabilities of CICs formation, we identified CD44, a well-known membrane protein associated with oncogenic phenotypes, as a negative regulator of CICs formation on tumor cells (Fig. 8). Targeting CD44 turned out to be an effective way to enhance CICs formation, immune killing, and suppression of tumor growth. Together, our work fits well with a novel therapeutic strategy of potentiating in-cell killing for cancer treatment.

The past decade had witnessed an ever-increasing interest in CICs investigation, which reached a broadly accepted concept that CICs, irrespective of the differences in working models and the underlying mechanisms, generally function to promote the death of those internalized cells in the acidified lysosome^33^, either apoptotic or non-apoptotic^1^. Though CICs-mediated cell death could be tumor-suppressive by limiting tumor cell growth or aneuploidy accumulation^14,19,34^, tumor cells were believed to take advantage of CICs to either feed themselves under stressed conditions^6,35^, or acquire genetic diversity and select fitter clones for further evolution^12,13,36^, or evade immune attacks via eliminating cytotoxic cells^5,37–39^. In contrast, we unexpectedly found that the penetration of NK cells could result in the death of the outer tumor hosts, indicating that the formation of CICs might serve as a cellular platform regulating the fates of both inner and outer cells. Consistent with this idea, the breast cancer cells were found to be able to cannibalize mesenchymal stem cells (MSC) to form CICs, leading to not only the death of inner MSC, but also the dormancy of the host breast cancer cells^40,41^. Meanwhile, the presence of internalized cells could serve as a physical barrier blocking the ingression of cleavage furrow during cytokinesis of outer cells, which eventually induced aneuploidy that conceivably promoted cancer progression^16,36^. Thus, future studies on CICs should not only focus on the inner cell fates, but may also pay attention to the outcomes of the outer cells. It should be noted that heterotypic CICs involving immune cells were rather various and complex in both cellular manifestations and molecular mechanisms, multiple models had been proposed including suicidal emperipolesis^37^, cannibalism^21^, phagopcytosis^42^, eclysis^43^, and emperitosis^5^ and the like^10,44^, therefore, their characteristics and outcomes should be analyzed in a case-based manner. As for the case in this study based on emperitosis model, CICs formation with NK cell penetration, instead of being an immune-suppressive mechanism, functions to promote immune killing of target tumor cells in a more efficient way. In fact, by revisiting previous studies based on this model, we found that some of them did identify the death of host cells associated with CICs formation, but failed to make a more informative investigation on it while focusing on the fates of the internalized cells^5,27,45^. This study may pave a novel way for further investigation of biological significance of other forms of heterotypic CICs.

Largely due to the lack of a reliable biochemical marker, the molecular regulation of the process of CICs formation was yet to be systemically investigated, which represents one of the major hurdles toward comprehensive characterization of CICs biology. One way out of this dilemma is to develop an artificial intelligence (AI)-based algorithm to identify CICs based on the microscopic images, thus enabling high throughput screening, which is still in its infancy^46^. Whereas as an alternative in this study, we managed to isolate isogenic cell clones that exhibit distinct abilities in forming heterotypic CICs from one parental cell line, which allowed us to systemically identify candidate genes potentially regulating CICs formation simply by expression profiling. It is conceivable that this method might also be utilized to decipher the molecular mechanisms underlying other types of CIC processes. Actually, we had tried to explore the molecular control of entotic CICs formation by the similar strategy, leading to the identification of a serial of functional regulators, such as PCDH7, IL-8^47,48^. With the same method, we identified CD44 on tumor cells as an important molecule that inhibits heterotypic CICs formation and in-cell killing, which was ascribed at least partially to its regulation on both cell–cell adhesion and host cell rigidity, two core elements identified to be essential for entotic CICs formation^18,49^. Interestingly, other than E-cadherin that mediates entotic CICs formation^14^, N-cadherin in complexed with β-catenin was found to be critical to mediate cell adhesion during the formation of heterotypic CICs in this study. CD44 functioned to downregulate the expression of N-cadherin and β-catenin, as well as to promote cytosolic localization of E-cadherin/γ-catenin, as a result, compromising intercellular adhesion and CICs formation. Moreover, in addition to be a signal receiver of extracellular stiffness^50^, CD44 was found also a positive regulator of cellular rigidity, that is, increased CD44 expression could significantly enhance cell stiffness while CD44 depletion resulted in more deformable cells as shown in Fig. 4. And this effect turned out to be coordinated by downstream RhoA and Rac1, two small GTPases that worked oppositely to control cellular stiffness and cell internalization as well, which, to our best knowledge, was demonstrated in heterotypic CICs formation for the first time. However, different from its positive role in entotic CICs formation, RhoA signaling functioned to inhibit the formation of heterotypic CICs though also via increasing cellular stiffness, which further supports that the molecular mechanisms of different CIC processes should be investigated in a context-dependent manner. By the way, the PLC/PRF/5 clones with more nuclei or those that were larger in size were more likely to internalize immune cells (data not shown), which is consistent with previous studies that syncytia by cell fusion efficiently internalized lymphocytes^38,51^.

The identification of CD44 as a negative regulator of in-cell killing on tumor cells provides an ideal target for therapeutic intervention. Treatments with NK cells were known to induce little graft versus host disease and cytokine release syndrome therefore was regarded as another promising strategy for cancer immunotherapy^52^. Nevertheless, the strategy remains to be optimized to increase their efficacies toward cancers, particularly the solid tumors, where the infiltration of NK cells, like other immune cells, was generally infrequent, leading to a low effector-to-target ratio (<1:1) that severely compromised the anti-tumor effects^53^. Whereas, our study showed that in-cell killing was much more potent than the traditional kiss-killing, which is particularly true for the condition of low effector-to-target ratio (≤2), where the in-cell killing maintained high killing efficacy of 33% while kiss-killing went down to 2% or so (Supplementary Fig. S1b). Thus, targeting CD44 to selectively activate in-cell killing may represent for a novel strategy of enhancing immune efficacy, an alternative to current strategies such as chimeric antigen receptor (CAR) or targeting immune checkpoint^54,55^. Moreover, targeting cancer stem cells (CSC), or cancer-initiating cells, was believed to be a curable way to eradicate cancers, and CD44 was one of the CSC markers for some cancers^29,56^. Thus, activating in-cell killing by anti-CD44 antibody that works together with either endogenous or infused exogenous NK cells may preferentially kill CSC for some types of cancers, reducing the frequency of relapse which warrants further investigation in the future.

In summary, this study reported in-cell killing as an unusual way for NK cells to kill their target cells, which is dependent on the formation of heterotypic CICs. Enhancing CIC formation by targeting the regulatory molecules such as CD44 turned out to be effective in enhancing in-cell killing and suppressing tumor growth, which may serve as a proof of concept study for developing novel anti-tumor strategies with further investigation of the underlying mechanisms. Besides, since heterotypic CICs could also form between different immune cells and normal cells^45,57^, further study on in-cell killing may shed light on the pathogenesis of certain immune-related processes, such as autoimmune diseases.

## Materials and methods

### Cells and culture conditions

Cell line PLC/PRF/5 and its derivatives were routinely maintained in Dulbecco’s modified Eagle’s medium (MACGENE Technology Ltd., Beijing, China) supplemented with 10% fetal bovine serum (Kangyuan Biology, China), and 100 units/mL penicillin plus 100 µg/mL streptomycin (Invitrogen). NK92MI cell line was maintained in RPMI 1640 medium (MACGENE Technology Ltd., Beijing, China) supplemented with 12.5% fetal bovine serum and 12.5% horse serum (Kangyuan Biology, China). Cell line CCRF-CEM and its derivative lines were maintained in RPMI 1640 medium supplemented with 10% fetal bovine serum, and 100 units/mL penicillin plus 100 µg/mL streptomycin. All cells were cultured in the humidified incubator of 5% CO_2_ at 37 °C.

### Antibodies and chemical reagents

Antibodies with working dilution, company source, and catalog number are listed as below: anti-CD44 (1:200 for IF or 1:1000 for WB; Abcam, #ab119863), anti-Hermes-1 (1:100; Bioxcell, #BE0262), anti-N-cadherin (1:200 for IF or 1:1000 for WB; BD Bioscience, #610921), anti-β-catenin (1:300 for IF or 1:1500 for WB; BD Biosciences, #610154), anti-E-cadherin (1:300 for IF or 1:1500 for WB; BD Biosciences, #610182), anti-γ-catenin (1:200 for IF or 1:1000 for WB; BD Biosciences, #610253), anti-RhoA (1:200 for IF or 1:1000 for WB; Cytoskeleton, #ARH03), anti-Rac1 (1:1000; Proteintech, #24072-1-AP), anti-pMLC (1:200 for IF or 1:1000 for WB; CST, #3671), anti-NKp46 (1:200 for IF; eBioscience, #11-3351-82). All the secondary antibodies (1:300 for IF and 1:3000 for WB) were purchased from Life technologies. Phalloidin (1:200) and Hoechst (1:2000) were purchased from Invitrogen. Z-ADD-CMK (Merck Millipore, #368050), LysoTracker® Red DND-99 (Invitrogen, #L7528) and Y27632 (MCE, # HY-10071) were purchased and used according to manufacturer’s instructions.

### Constructs

pQCXIP-CD44-eGFP-N1 was cloned by inserting the CD44 coding sequence into the *Xho* I/*Eco*R I site of the pQCXIP-eGFP-N1 vector. pEGFP -β-catenin-C1 was a gift from W. James Nelson from Stanford University. pQCXIP-N-cadherin-mCherry was stored in the laboratory. pQCXIP-mCherry-RhoA and pEGFP-Rac1-WT were gifts from Dr. Alan Hall (MSKCC, USA).

### Virus production and infection

To establish stable expression cell lines, target genes together with lentiviral or retroviral constructs and packaging plasmids were transfected into HEK293FT cells by Lipofectamine 2000 reagent (Invitrogen) as described^58^. Target cells were transduced with 1 mL viral supernatant mixed with 8 μg/mL polybrene (Sigma) for 6 h followed by regular medium. Virus-infected cells were selected and grown in the medium with 1 µg/mL puromycin or 400 µg/mL G418.

### Immunostaining and immunoblotting

Immunostaining was performed as previously described^59^ with the following steps: cells were fixed with 4% paraformaldehyde for 10 min at room temperature, then permeabilized with 0.2% Triton X-100/PBS for 3 min, and washed with PBS followed by blocking with 5% BSA for 1 h. The fixed samples were incubated with primary antibodies dissolved in 5% BSA at 4 °C overnight, washed with PBS three times, and incubated with secondary antibodies for 1 h at room temperature. After washing with PBS three times, samples were mounted with mounting medium (ZSGB-BIO, #ZLI-9557) and imaged by Nikon ECLIPSE Ti-U epi-fluorescence microscope. Immunoblotting was performed as previously described^60^ with the following steps: total proteins were extracted and quantified with Pierce BCA Protein Assay KIT (Thermo, #23227), then subjected to SDS-PAGE and transferred to polyvinylidene fluoride (PVDF) membranes. The transferred samples were blocked with 5% bovine serum albumin (BSA) and probed with primary antibodies and species matched secondary antibodies, then visualized by chemiluminescence SuperSignal KIT (Thermo, #34095) according to the manufacturer’s instructions.

### Tissue microarray (TMA) staining and image processing

A liver cancer TMA slide (HLiv-HCC180Sur-04), purchased from SHANGHAI OUTDO BIOTECH CO. LTD, was stained with antibodies against CD44, NKp46, and E-cadherin, and scanned by the Vectra® Polaris™ automated quantitative pathology imaging system (Perkin Elmer) as previously described^61^. The cellular membrane was labeled by E-cadherin and NK cells were indicated by NKp46. The expression level of CD44 was indicated as 1, 2, 3, 4 from low to high (Fig. 7c), and CD44 expression of each tissue core was categorized to one number. The cell-in-cell frequency = the number of internalized NK cells marked by NKp46 (Fig. 7f) in each tissue core. TNM stage information (Supplementary Table S1) was provided together with the TMA slide by SHANGHAI OUTDO BIOTECH CO. LTD. The diameter of each core was 1.5 mm. All tissues were collected under the highest ethical standards with the donor being informed completely and with their consent.

### siRNA transfection and transcription-quantitative PCR (RT-PCR)

The RNA interference hairpin constructs for human CD44, N-cadherin, β-catenin, RhoA, and Rac1 were purchased from the Chinese company GenePharma. For siRNA transfection (sequences showed in key resources table), 2.0 × 10^5^ PLC/PRF/5 cells were transfected with 50 nM siRNA using Lipofectamine RNAiMAX Reagent (Invitrogen, #13778-150). Total RNA was extracted from siRNA transfected cells 48 h post-transfection, and reverse transcription-PCR was performed with TransScript one-step gDNA Removal and cDNA Synthesis SuperMix (TransGen Biotech, #AT311-02) for the preparation of cDNA. Quantitative RT-PCR was performed routinely using target primers for the amplification of the coding region of the target gene using SYBR Green Realtime PCR Master Mix (TOYOBO, #QPK-201).

### Cell cluster assays

To assess cell–cell adhesion ability gathering into clusters, 3 × 10^5^ PLC/PRF/5 cells were suspended above 0.5% concreted agarose for 6 h, which was bedded in 6-well plates. Then cell clusters were collected gently and spun onto glass slides at 400 rpm for 3 min by a spinning machine (Low-speed tabletop centrifuge DT5-6, ERA BEILI CENTRIFUGE CO. China). The cell clusters on glass slides were stained with DAPI and photographed by NIKON microscope. Cells in cluster rate (%) = (number of cells involved in the cluster/number of total cells) × 100. We defined the cluster as a collection of cells adhered to each other which contains >6 cells.

### Heterotypic CIC formation assay

To induce heterotypic CIC formation, about 1 × 10^4^ PLC/PRF/5 cells were cultured in 12-well plates overnight, and 5 × 10^4^ immune cells were seeded into the plates and co-cultured for 8 h. Then the suspended immune cells were washed out with PBS and the adherent PLC/PRF/5 cells were trypsinized and spun on glass slides by centrifugation at 400 rpm for 3 min. After being fixed with 4% paraformaldehyde, the cells on glass slides were stained with DAPI and photographed by an NIKON microscope. The cell-in-cell 1 (%) = numbers of outer PLC/PRF/5 cells in CICs/number of total PLC/PRF/5 cells counted. The cell-in-cell 2 (%) = numbers of internalized CCRF cells/number of total PLC/PRF/5 cells counted. Structures with the inner cells being fully enclosed by outer cells were counted.

### Quantification of winner/loser cell identity

For identity analysis of winners and losers in homotypic CIC structures, the same amounts of cells from two cell lines were labeled with different fluorescent dyes and then co-cultured in suspension on the 0.5% agarose gel for 6 h, and then spun onto glass slides followed by staining with DAPI and photographed by NIKON microscope. Cell structures with more than half of the cell body internalized were counted as CICs. We defined the inner cells as the loser and the outer as the winner in a homotypic CIC structure.

### Agarose compression assay

Agarose compression assay was performed as described^28^. Briefly, a piece of transparent agarose gel (2% weight) was cast into a 200 μm thick sheet by pouring hot agarose solution between two glass plates separated by 0.2 mm coverslips and cooled at room temperature. The suspended cells on the culture dish with glass bottom were covered with a piece of agarose gel and compressed after taking away the culture medium. Then compressed cells were captured in DIC and fluorescence channels and scanned by Z stacks every 1 min with 40× objective lenses at 37 °C and 5% CO_2_ for 1 h by UltraVIEW VoX Spinning Disk Confocal microscope, and then analyzed by NIS-Elements F 3.0 software (Nikon, Japan). The average pressure on one single cell is calculated by the equation *W* * *L* * *R* * *R* * *M* * *g*/(*S* * *N*), *W* is the width of the picture in the microscope, *L* is the length of the picture, *R* is the microscopy coefficient, *M* is the weight of agarose gel, *g* = 9.8 N/kg, *S* is the size of agarose gel, *M* * *g*/*S* is the gravity density, *N* is the number of cells in the window.

### Immune killing in vitro

To explore the killing efficiency of immune cells to PLC/PRF/5 cells, about 1 × 10^4^ PLC/PRF/5 cells were cultured in 12-well plates overnight, and 1 × 10^4^ NK92MI cells or PBMC were seeded into the plates and co-cultured for 24 h. Then the suspended immune cells were washed out with PBS and the adherent PLC/PRF/5 cells were trypsinized and counted by hemocytometer, and the survival ratio = the number of NK92MI-treated PLC/PRF/5/the number of control PLC/PRF/5. The second quantification method was by way of CCK8 incorporation, the PLC/PRF/5 cells, NK92MI cell, PLC/PRF/5 cells co-cultured with NK92MI cells were cultured in the presence of CCK8 reagents for 10 min before being subjected to measurement at the wavelength of 450 nm by a microplate reader. The survival ratio = (OD450 value of PLC/PRF/5 cells–OD450 value of NK92MI cells) /OD450 value of PLC/PRF/5 cells co-cultured with NK92MI cells. The killing ratio = 1—the survival ratio. These two statistical ways get consistent results.

### Xenograft assay

#### SCID Beige was a type of severe combined immunodeficiency mouse that has barely immune cells

For tumor prevention model on Supplementary Fig. S7a–d, the F6ft or A4S-CD44 cells (1 × 10^7^ per side injection) were randomized into four groups and mixed with rat IgG (50 μg per side injection), anti-CD44 mAbs (50 μg per side injection), rat IgG (50 μg per side injection) and NK92MI cells (5 × 10^6^ per side injection), anti-CD44 mAbs (5 μg per side injection) and NK92MI cells (5 × 10^6^ per side injection), respectively. Then these mixtures were implanted in female 6-week-old SCID Beige mice. Mice were monitored for tumor growth throughout the process till the 21st day to collect tumors.

Tumors were collected and processed for paraffin embedding. Sections of xenograft were immune-stained with antibodies against β-catenin (1:200; BD Bioscience, # 612154) and NKp46 (1:200; eBioscience, #11-3351-82). Images were taken in four channels (FITC, TRITC, CY5, and DAPI) by Nikon microscopy and analyzed for CIC formation between shNC-F6ft cells and NK92MI cells.

#### Transmission electron microscopy

Cells were fixed with 2.5% (vol/vol) glutaraldehyde with Phosphate Buffer (PB) (0.1 M, pH 7.4), washed four times in PBS at 4 °C. Then cells were post-fixed with 1% (wt/vol) OsO_4_ and 1.5% (wt/vol) potassium ferricyanide aqueous solution at 4 °C for 2 h, dehydrated through a graded ethanol series (30%, 50%, 70%, 80%, 90%, 100%, 100%, 5 min each at 4 °C) into pure acetone (2 × 5 min). Samples were infiltrated in graded mixtures (3:1, 1:1, 1:3) of acetone and SPI-PON812 resin (16.2 mL SPI-PON812, 10 mL DDSA, and 8.9 mL NMA), then changed to pure resin. Finally, cells were embedded in pure resin with 1.5% BDMA and polymerized for 12 h at 45 °C, 48 h at 60 °C. The ultrathin sections (70 nm thick) were sectioned with a microtome (Leica EM UC6), double-stained by uranyl acetate and lead citrate, and examined by a transmission electron microscope (FEI Tecnai Spirit 120 kV).

### Statistics

Data were presented as means ± SD. *P* < 0.05 was considered statistically significant, which was calculated by a two-tailed Student’s *t*-test or Dunnett *t*-test using Excel or GraphPad Prism software. * represents for *P* < 0.05; ** for *P* < 0.01; *** for *P* < 0.001; **** for *P* < 0.0001.

## Supplementary information

Supplementary Information

## Data Availability

This study produced and analyzed the following dataset: Oncomine (https://www.oncomine.org/resource/login.html#), GEPIA (http://gepia.cancer-pku.cn/detail.php?gene=&clicktag=survival), Kaplan–Meier Plotter (http://kmplot.com/analysis/index.php?p=service&start=1).
