# Supplementary material for: Cell-in-cell structure mediates in-cell killing suppressed by CD44
Source: Cell Discov. 2022 Apr 19;8:35. doi: 10.1038/s41421-022-00387-1 (PMC9016064; doi:10.1038/s41421-022-00387-1)
Supplement: Supplementary file 1 — Supplementary Information [file 41421_2022_387_MOESM1_ESM.pdf]

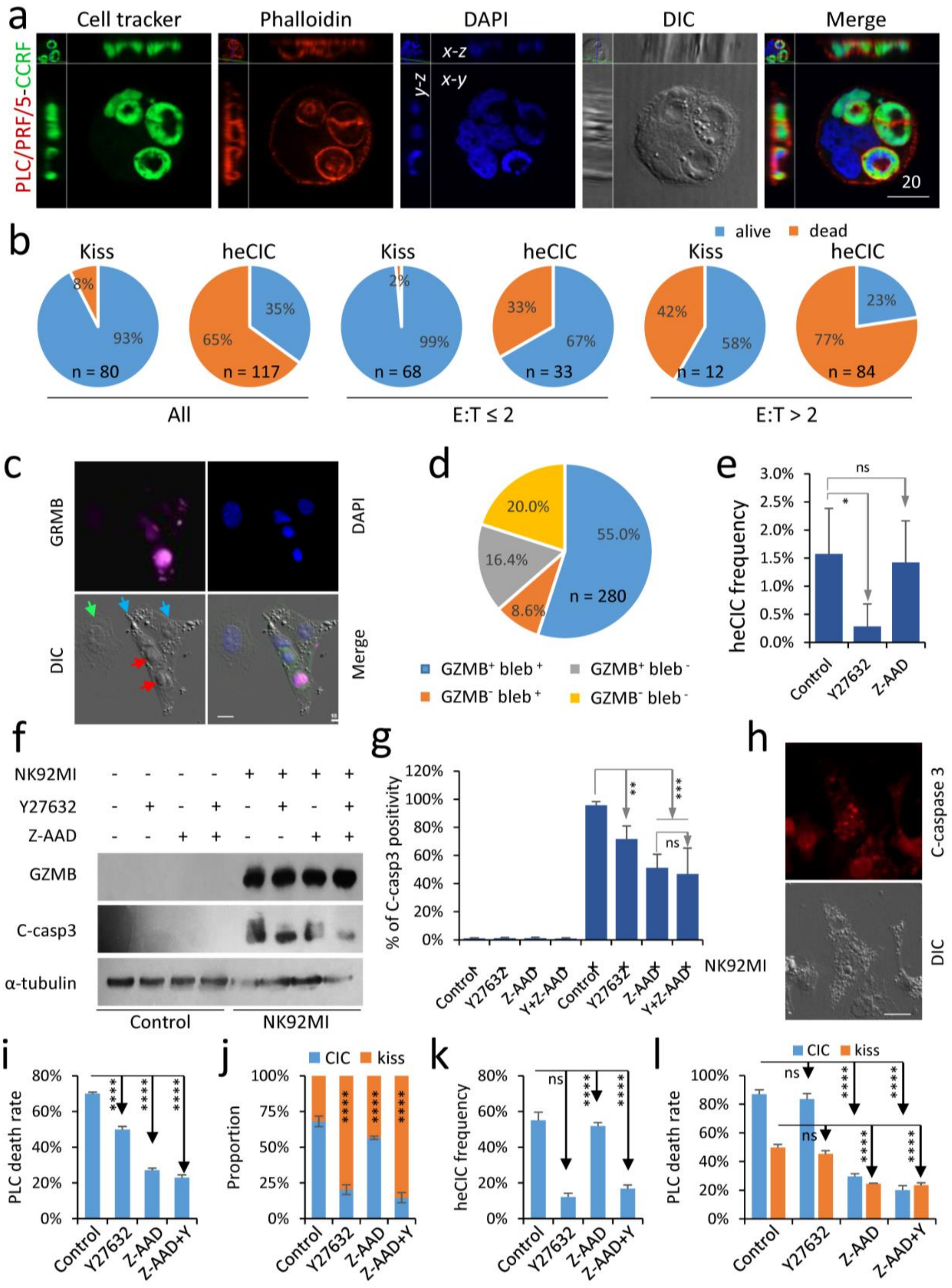

Supplementary Fig. S1 The heCIC-mediated in-cell killing was dependent on GZMB. Related to Fig. 1.

a Representative images in different channels for the heCIC structure formed between PLC/PRF/5 (phalloidin-red) and CCRF-CEM cells (EGFP-green). Scale bar, 20 μm. **b** Fate analysis of PLC/PRF/5 cells that were in contact with NK92MI cells from outside (kiss), or internalized NK92MI cells (CIC), by time lapse imaging of 24 hours. The ratios of effector cells (E for NK92MI): target cells (T for PLC/PRF/5) were indicated. **c** Representative images for granzyme B (GZMB) staining. Red arrows indicate two NK92MI cells internalized by a PLC/PRF/5 cell. Blue arrows indicate two PLC/PRF/5 cells that are positive in GZMB and blebs. Green arrow indicates one PLC/PRF/5 cell that is negative in GZMB and blebs. Scale bar, 10 μm. **d** Quantification of GZMB positivity and blebs in PLC/PRF/5 cells co-cultured with NK92MI cells for 24 hours, n = 280. **e** The formation of heCIC structures between NK92MI and PLC/PRF/5 (E : T = 1 : 1) was inhibited by Y27632 (10 μM), but not Z-AAD-CMK (50 μM), as quantified on cytospin images. n > 600 cells/each, ns: not significant, * $p < 0.05$. **f, g** Inhibition of either GZMB by Z-AAD-CMK (50 μM) or heCIC formation by Y27632 (10 μM) could compromise the activation of caspase-3 in PLC/PRF/5 co-cultured with NK92MI cells for 24 hours, as evidenced by reduced processing of caspase-3 by Western blot (**f**) and reduced positivity in cleaved-caspase-3 by immunostaining (**g**). ** $p < 0.01$; *** $p < 0.001$. **h** Representative images for staining of cleaved-caspase 3 in PLC/PRF/5 cells. Scale bar, 20 μm. **i-k** The percentage of dead PLC/PRF/5 cells (**i**), the proportion of two death modes (**j**) and the formation of heCIC structures (**k**) in 24-hour coculture with NK92MI cells (E : T = 1 : 1) in the presence of Y27632 (10 μM), or Z-AAD-CMK (50 μM), or Y27632 plus Z-AAD-CMK, respectively, as quantified by time-lapse imaging. Death rate = dead PLC cells / all PLC cells (**i**), proportion of CIC-death = death by CIC / (death by CIC + death by kiss) (**j**), proportion of kiss-death = death by kiss / (death by CIC + death by kiss) (**j**), heCIC frequency = PLC cells involved in heCIC structure / all PLC cells (**k**). Data are mean ± SD of three images of 20 x objective. n > 100 cells /or structures analyzed for each treatment. ns: $p > 0.05$; *** $p < 0.001$; **** $p < 0.0001$. **l** The percentage of dead PLC/PRF/5 cells that formed heCIC structures with NK92MI cells or were contacted/kissed by NK92MI cells (**l**) in 24-hour coculture with NK92MI cells (E : T = 1 : 1) in the presence of Y27632 (10 μM), or Z-AAD-CMK (50 μM), or Y27632 plus Z-AAD-CMK, respectively, quantified by time-lapse imaging. Death rate of CIC = dead PLC cells in heCIC structure / all PLC cells in heCIC structure, Death rate of kiss = dead PLC cells kissed by NK92MI cells / all PLC cells kissed by NK92MI cells. Data are mean ± SD of three images of 20 x objective. n > 100 cells /or structures analyzed for each treatment. ns: $p > 0.05$; **** $p < 0.0001$.

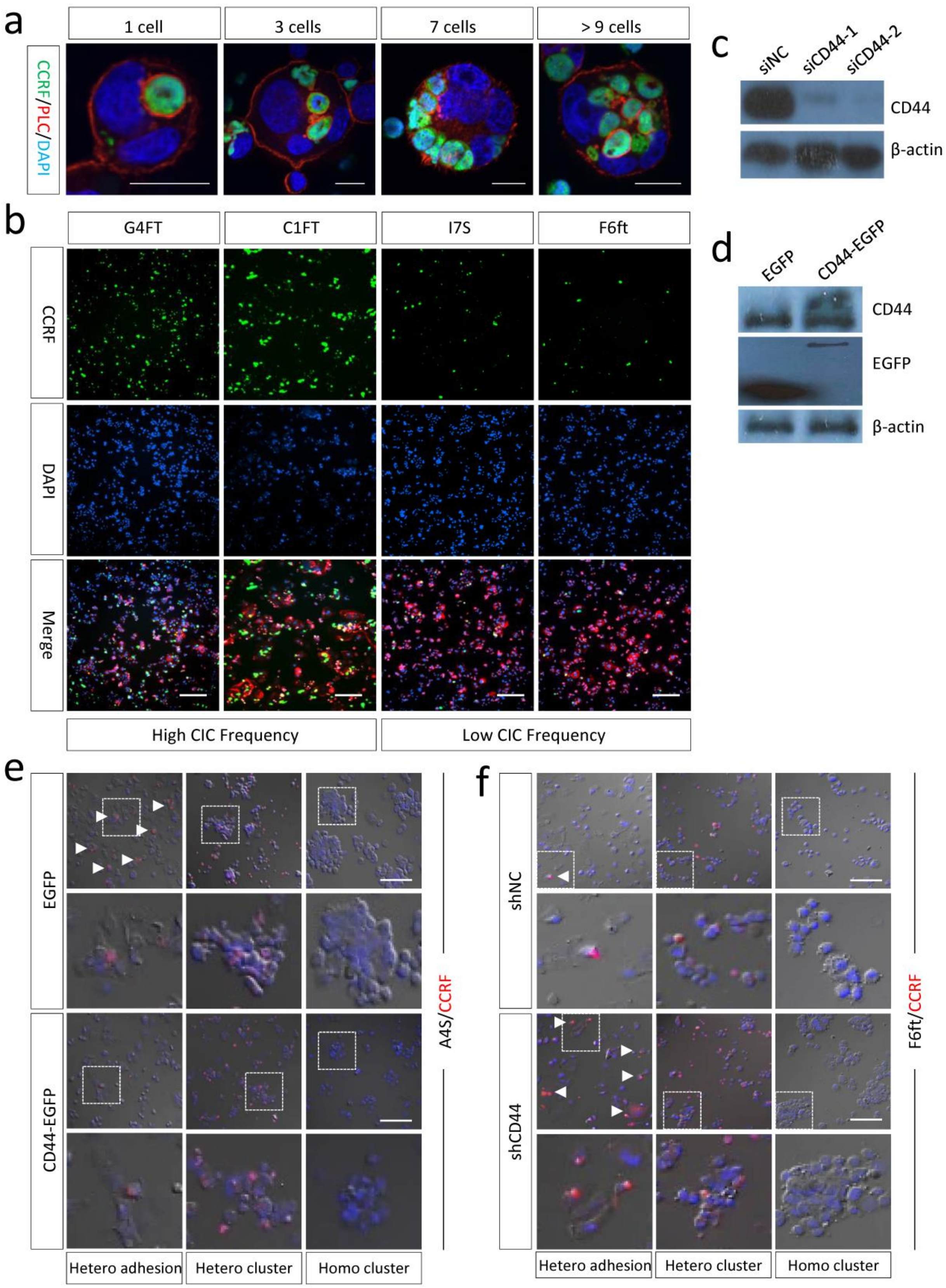

Supplementary Fig. S2 CD44 as a negative regulator of heCIC formation. Related to Fig. 2.

a Representative images of heCIC structures with different number of CCRF-CEM cells (EGFP-green) internalized by PLC/PRF/5 cell (phalloidin-red). Scale bar, 20 μm. **b** Representative images in channels for different number of CCRF-CEM cells (EGFP-green) internalized by PLC/PRF/5 cell (phalloidin-red) of high heCIC frequency (G4FT and C1FT) or low heCIC frequency (I7S and F6ft). Scale bar, 50 μm. **c, d** CD44 expression upon siRNA-mediated knockdown in F6ft cells (**c**) or overexpression in A4S cells (**d**) as detected by Western blot. **e, f** Representative images of heterotypic intercellular adhesion (hetero adhesion), heterotypic cell clustering (hetero cluster) formed between CCRF-CEM (red) and A4S cells (**e**) or F6ft cells (**f**), and homotypic cell clustering (homo cluster) formed between isogenic tumor cells. The cell cluster was defined as a cell colony that contains more than 6 cells. Zoomed-in panels showed cropped view of the white boxed region. White arrowhead indicates heterotypic adhesion. Scale bar = 50 μm.

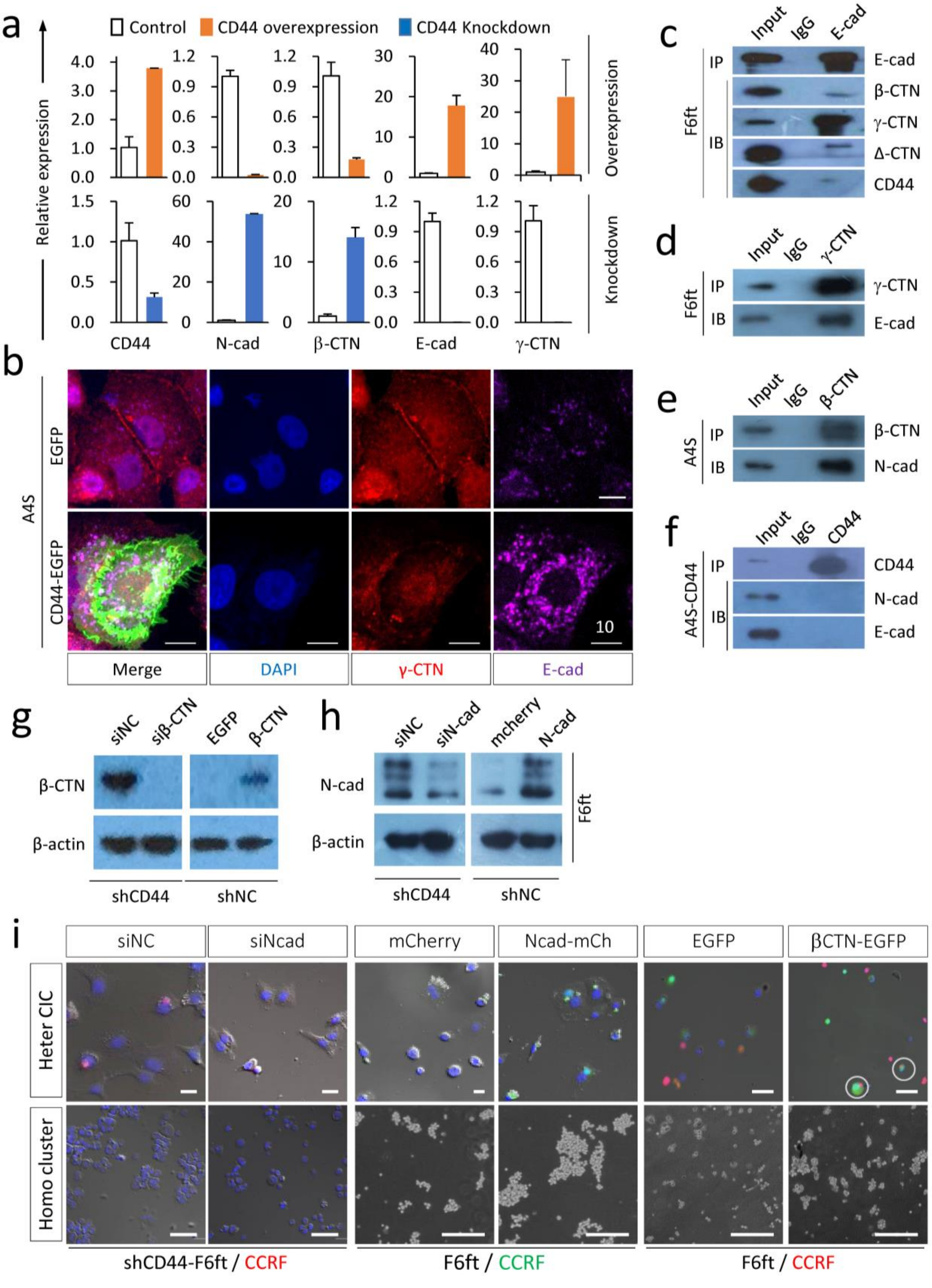

Supplementary Fig. S3 CD44 suppressed cellular adhesion. Related to Fig. 3.

a The relative mRNA expression of adhesion molecules upon CD44 knockdown or overexpression as

detected by RT-PCR. Data are mean ± SD of triplicate experiments. **b** Confocal images showed subcellular localization of γ-catenin and E-cadherin in control and CD44-EGFP expressing A4S cells. Scale Bar, 10 μm. **c, d** E-cadherin is in complex with γ-catenin as determined by co-immunoprecipitation analysis in F6ft cells. **e** N-cadherin is in complex with β-catenin as determined by co-immunoprecipitation analysis in A4S cells. **f** No interaction was detected between CD44 and N-cadherin, or E-cadherin in A4S cells overexpressing CD44. **g, h** Expression of β-catenin (**g**) and N-cadherin (**h**) in F6ft cells analyzed by Western blot. **i** Images showed the heCIC structures formed between CCRF-CEM and F6ft cells, and the homotypic clusters of F6ft cells with the knockdown or overexpression of N-cadherin or β-catenin.

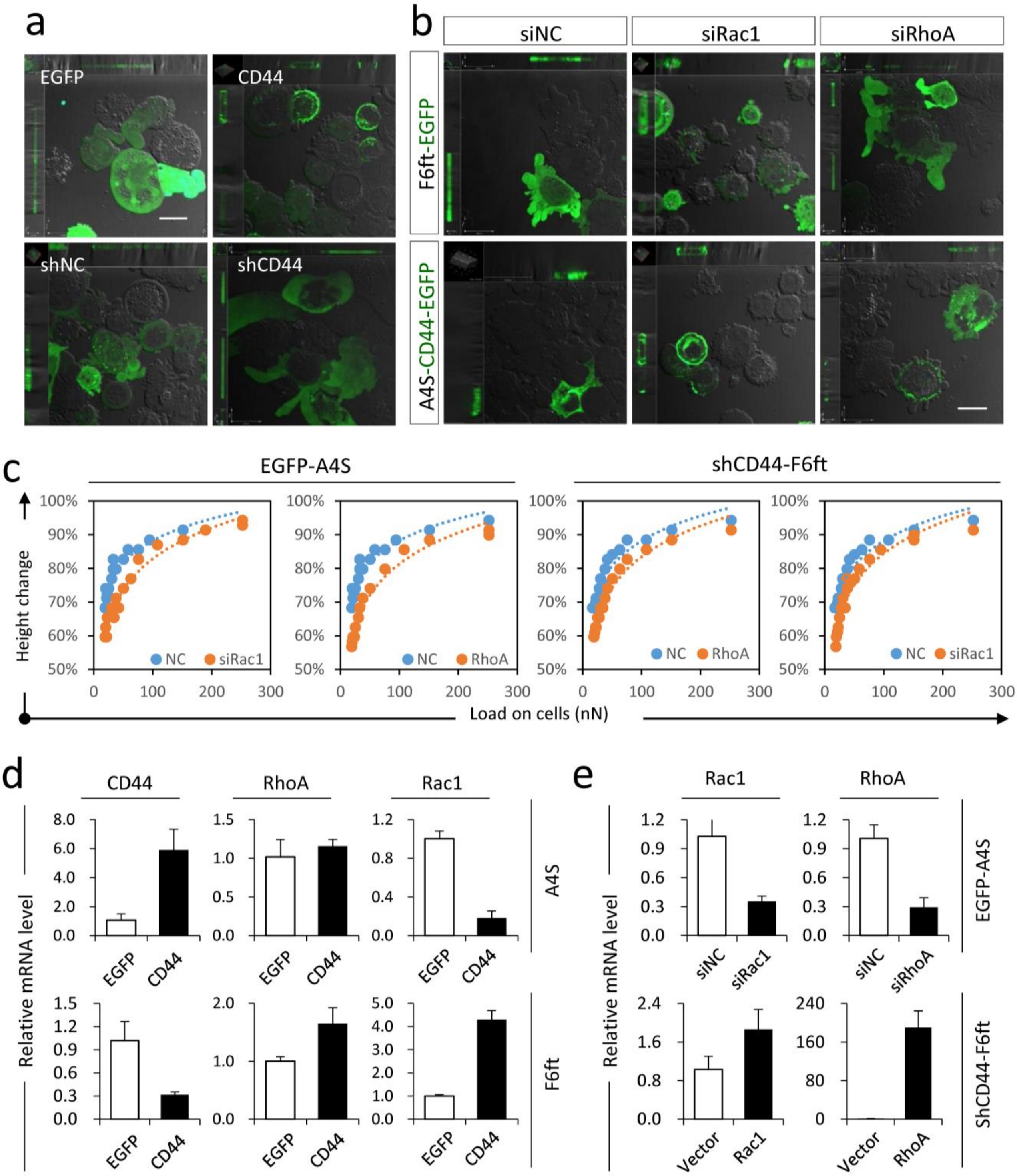

Supplementary Fig. S4 The PLC/PRF/5 isogenic cells were compressed and deformed by agarose gel overlay.

a, b Images show the deformed cells overlaid with the agarose gel of same gravity density. **c** Overexpressing RhoA or knocking down Rac1 enhance cell rigidity in cells with either CD44 low-expression (EGFP-A4S) or depletion (shCD44-F6ft) as determined by agarose compression assay. **d,**

e Expression of CD44, RhoA and Rac1 upon overexpression or knockdown as determined by quantitative RT-PCR.

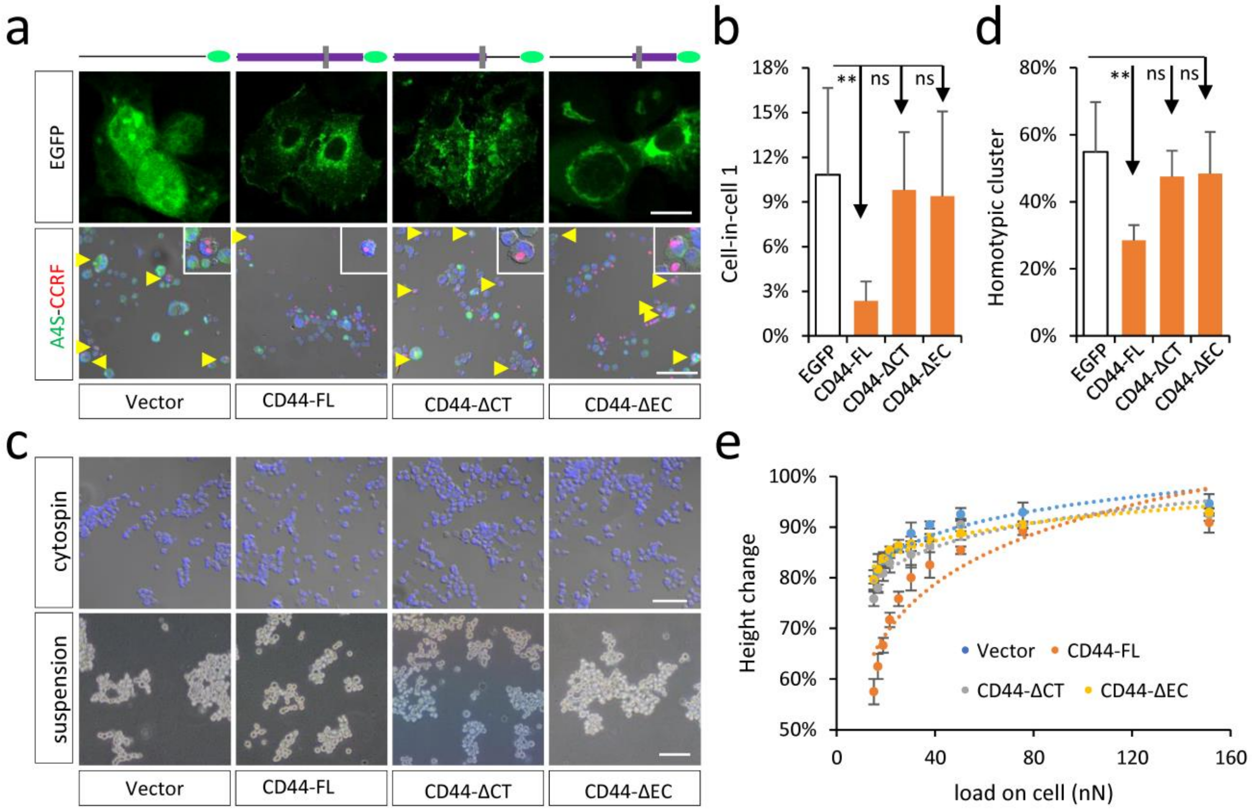

Supplementary Fig. S5 Truncation analysis of CD44 molecule.

a Representative images for subcellular localization and heCIC formation upon CD44 truncation as indicated. FL: full length, ΔCT: C-terminal truncation, ΔNT: N-terminal truncation. Scale bar, 10 μm and 50 μm, respectively. **b** The frequencies of heCIC formation in CD44 mutants-overexpressed A4S cells co-cultured with CCRF-CEM cells for 8 hours. n > 500 cells for each cell line. At least five images of 20x objective were quantified for each cell line. Ns: no statistical difference; ** $p < 0.01$. **c, d** Representative images (**c**) and frequencies (**d**) of homotypic cluster in CD44 mutants-overexpressed A4S cells. Scale bar, 50 μm and 50 μm, respectively. n > 800 cells for each cell line. At least five images of 20x objective were quantified for each cell line. Ns: no statistical difference; ** $p < 0.01$. **e** Cellular height changes of A4S cells overexpressing CD44 mutants in responding to different weight loads (nN) as determined by agarose compression assay. Data are mean ± SD of triplicate experiments.

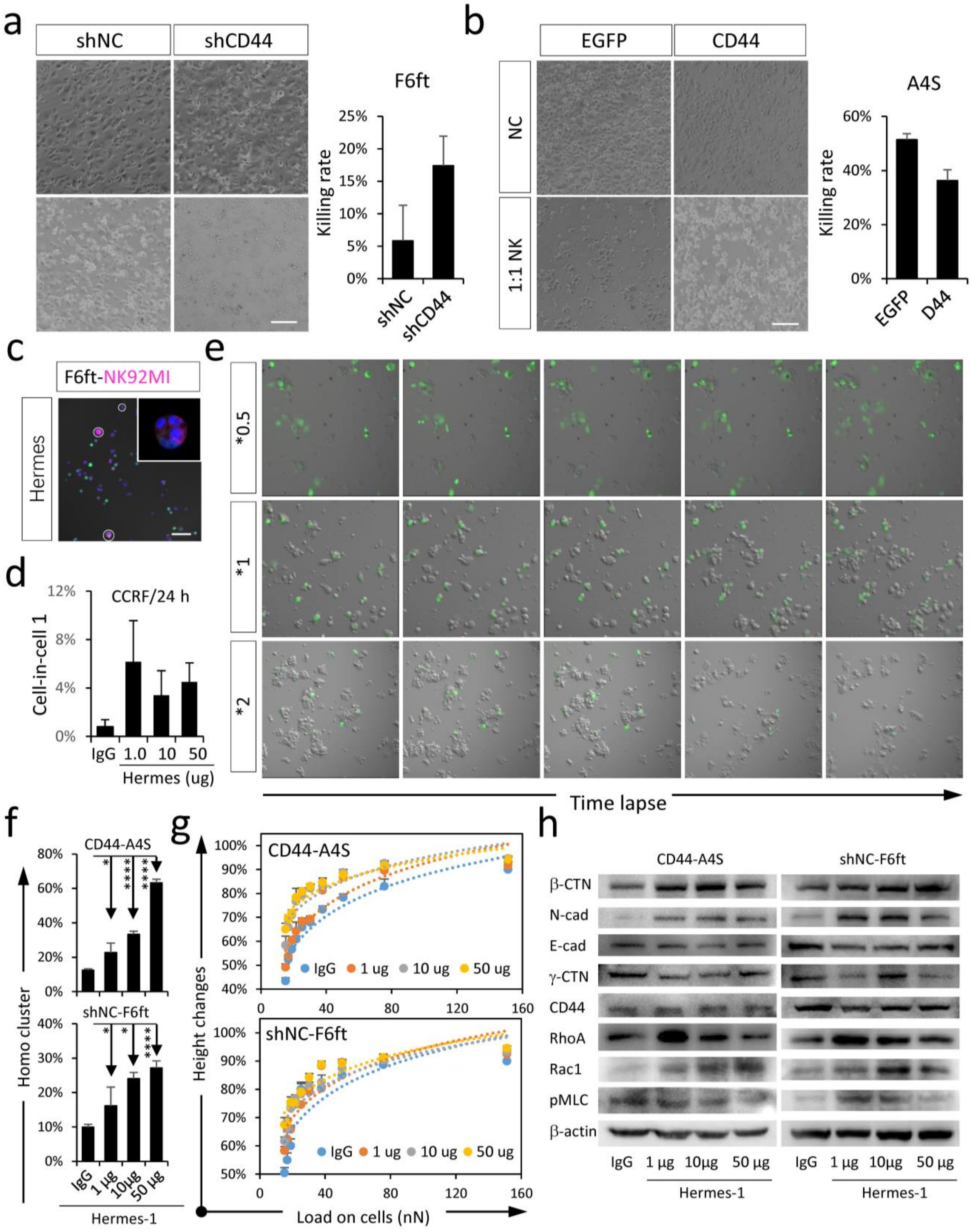

Supplementary Fig. S6 The CD44-mediated immune killing was associated with heCIC.

a, b CD44 knockdown (**a**) or overexpression (**b**) enhanced or suppressed tumor cell killing by NK92MI cells in a co-culture of 24 hours at an E/T ratio of 1:1. Representative images and quantification graphs

are showed. For each quantification: n > 300, $p < 0.05$. Scale bar: 50 μm. **c** Representative images of heCIC structure formed between NK92MI (NKp46, purple) and F6ft cells when the co-culture was treated with 10 μg Hermes-1 for 8 hours. **d** Quantification of heCIC formed between F6ft and CCRF-CEM cells in the presence of different amounts of Hermes-1. n > 300, $p < 0.05$ between control (IgG) and Hermes-1-treated groups. **e** The survival of PLC/PRF/5 cells (green) in the presence of different amounts of NK92MI cells (E/T=0.5, 1, 2, respectively) as showed by still images from a time-lapse imaging of 24 hours. **f** The frequency of homotypic cluster formed by A4S cells overexpressing CD44 (CD44-A4S) or F6ft cells transfected with vector (shNC-F6ft) in the presence of different amounts of Hermes-1 for 24 hours (pretreated for 16 hours and present for 8 hours during cluster assay). Data are mean ± SD of more than three images of 20 x objective. n > 300 cells analyzed for each. * $p < 0.05$; **** $p < 0.0001$. **g** Hermes-1 treatment enhanced cellular deformation in responding to different weight loads (nN) as determined by agarose compression assay. Data are mean ± SD of triplicate experiments. **h** Expression of CIC-related molecules upon treatment of different amounts of Hermes-1 for 24 hours by Western blot. The β-actin was used as a loading control.

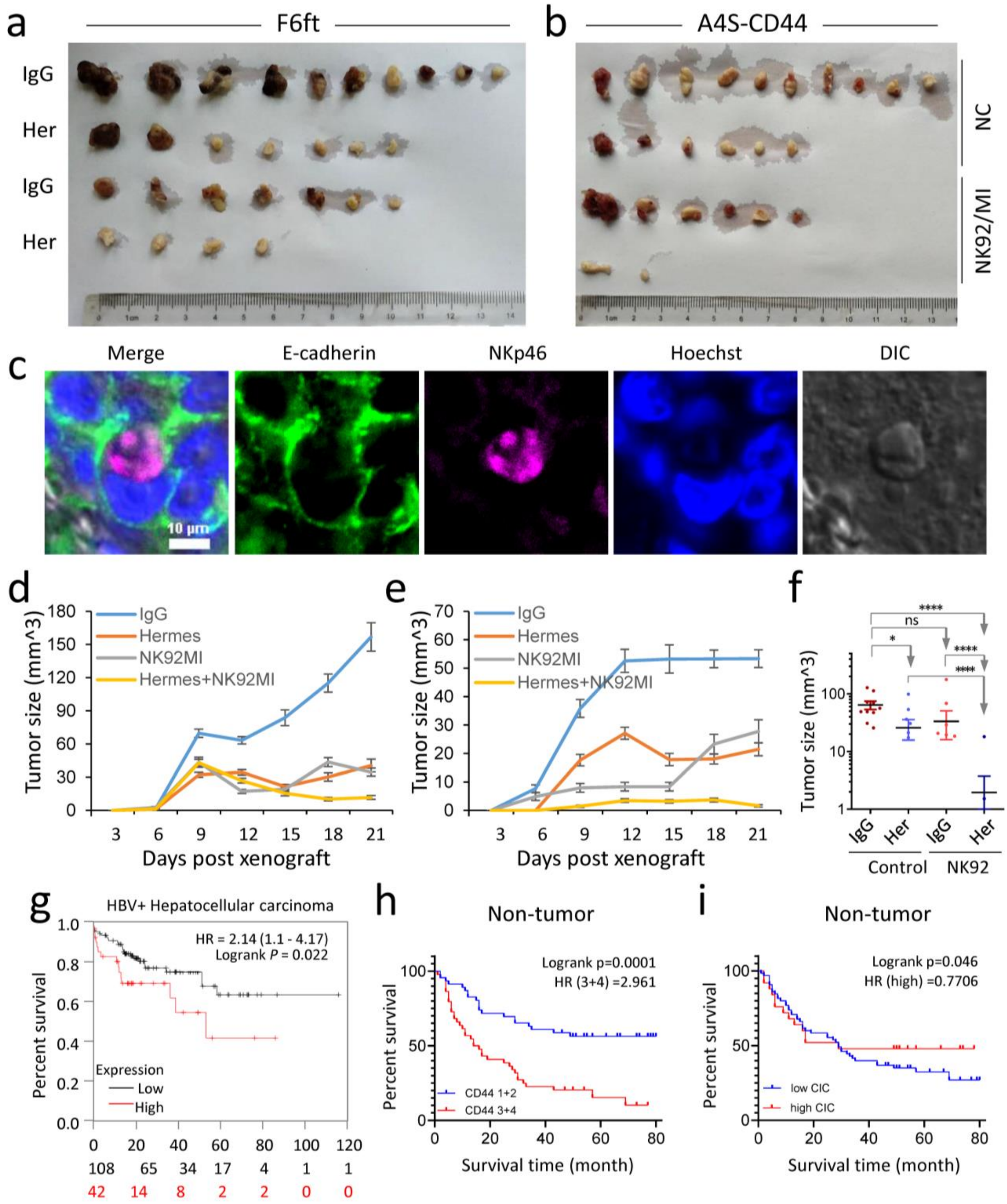

Supplementary Fig. S7 High expression of CD44 promoted tumor growth.

a, b Pictures for xenografts of F6ft cells (**a**) and A4S cells overexpressing CD44 (A4S-CD44) (**b**) together with Hermes-1 antibody and/or NK92MI cells (1:1), tumors were collected 21 days post implantation. **c** Channeled representative images of heCIC structures formed between F6ft cells and NK92MI cells in xenograft tumor tissues stained with antibodies against E-cadherin (green) and

NKp46 (red). Scale bar, 10 µm. **d, e** Tumor size graph for xenografts of F6ft cells (**d**) and A4S cells overexpressing CD44 (A4S-CD44) (**e**) together with Hermes-1 antibody and/or NK92MI cells (0.5:1), tumors were collected 21 days post implantation. Standard error (SE) was employed. **f** Quantification of xenografted tumors 21 days post the inoculation of A4S-CD44 cells together with the indicated antibodies and NK92MI (NK92) cells. Her: Hermes-1. ns: not significant; * $p < 0.05$; **** $p < 0.0001$. F test was employed. **g** Kaplan-Merier survival curves showed that high expression of CD44 was associated with a shorter survival period for patient with HBV positive hepatocellular carcinoma as analyzed by the KP plotter. **h, i** Survival analysis of patients with hepatocellular carcinoma stratified by CD44 expression (**h**) or CIC level (**i**) in non-tumor tissues by Kaplan-Merier analysis.
